# Spatiotemporal multi-scale modeling of radiopharmaceutical distributions in vascularized solid tumors

**DOI:** 10.1038/s41598-022-18723-6

**Published:** 2022-08-26

**Authors:** Mohammad Kiani Shahvandi, M. Soltani, Farshad Moradi Kashkooli, Babak Saboury, Arman Rahmim

**Affiliations:** 1 Department of Mechanical Engineering, K. N. Toosi University of Technology, Tehran, Iran; 2 Department of Electrical and Computer Engineering, University of Waterloo, Waterloo, ON Canada; 3 Centre for Biotechnology and Bioengineering (CBB), University of Waterloo, Waterloo, ON Canada; 4 Advanced Bioengineering Initiative Center, Multidisciplinary International Complex, K. N. Toosi University of Technology, Tehran, Iran; 5 Department of Radiology, Hospital of the University of Pennsylvania, 3400 Spruce Street, Philadelphia, PA 19104 USA; 6 Department of Integrative Oncology, BC Cancer Research Institute, Vancouver, BC Canada; 7 Departments of Radiology and Physics, University of British Columbia, Vancouver, BC Canada

**Keywords:** Computational models, Computational biophysics, Cancer imaging, Cancer microenvironment, Cancer models, Tumour angiogenesis, Tumour biomarkers, Tumour heterogeneity

## Abstract

We present comprehensive mathematical modeling of radiopharmaceutical spatiotemporal distributions within vascularized solid tumors. The novelty of the presented model is at mathematical level. From the mathematical viewpoint, we provide a general modeling framework for the process of radiopharmaceutical distribution in the tumor microenvironment to enable an analysis of the effect of various tumor-related parameters on the distribution of different radiopharmaceuticals. We argue that partial differential equations (PDEs), beyond conventional methods, including ODE-based kinetic compartment modeling, can be used to evaluate radiopharmaceutical distribution in both time and space. In addition, we consider the spatially-variable dynamic structure of tumor microvascular networks to simulate blood flow distribution. To examine the robustness of the model, the effects of microvessel density (MVD) and tumor size, as two important factors in tumor prognosis, on the radiopharmaceutical distribution within the tumor are investigated over time (in the present work, we focus on the radiopharmaceutical [^18^F]FDG, yet the framework is broadly applicable to radiopharmaceuticals). Results demonstrate that the maximum total uptake of [^18^F]FDG at all time frames occurs in the tumor area due to the high capillary permeability and lack of a functional lymphatic system. As the MVD of networks increases, the mean total uptake in the tumor is also enhanced, where the rate of diffusion from vessel to tissue has the highest contribution and the rate of convection transport has the lowest contribution. The results of this study can be used to better investigate various phenomena and bridge a gap among cancer biology, mathematical oncology, medical physics, and radiology.

## Introduction

Tumor heterogeneity is among the most important contributing factors to cancer treatment failure^1–5^. *Precision oncology* aims to address this issue by investigating tumor biology variations and tumor micro-environment (TME) heterogeneity. Personalized treatment planning is required to mitigate the effect of these variations, away from a one-fits-all approach. The theranostic paradigm is one of the best platforms to actualize *precision oncology*^6–9^. Its therapeutic aspect, through radiopharmaceutical therapy, targets specific tumor cells while its diagnostic counterpart helps to illustrate tumor heterogeneity. This combination is a perfect match for personalized cancer care. In this paradigm, similar radiopharmaceuticals are used for diagnosis and therapy. Radiopharmaceuticals are labeled with two categories of radionuclides, one for diagnostic purposes and the other for treatment. The diagnostic radionuclide usually is a positron emitter (such as ^18^F, ^13^ N, ^11^C, or ^15^O), while the therapeutic radionuclide is an alpha or beta emitter (such as ^225^Ac, ^177^Lu, or ^90^Y). The critical property of these two radiopharmaceuticals (i.e., diagnostic and therapeutic) is their pharmacokinetic similarities. This phenomenon will provide physicians with a unique opportunity to image the tumor heterogeneity on the one hand and to address this depicted heterogeneity through therapy adjustment on the other.

Tumor heterogeneity has two components: tumor cell variations (inter-tumoral and intra-tumoral mutations) and tumor micro-environment heterogeneity (including vascularity, permeability and diffusability, hypoxia, and the extend of immune system presence and function, among the others)^10–12^. Quantitative imaging using dynamic positron emission tomography (PET) represents the aggregation of these two factors. Traditionally, the second category (tumor micro-environment) was assumed insignificant, and the quantitative models did not consider spatial heterogeneity of the micro-environment. This assumption appears unrealistic in light of recent discoveries about the pivotal role of the tumor micro-environment in treatment success and failure^13,14^. To address this unmet and critical need, the conventional temporal models (such as compartmental kinetic analysis based on ordinary differential equations (ODEs)^15,16^) should be updated using spatiotemporal distribution models (SDMs) based on partial differential equations (PDEs))^17–24^. This study aimed to model one of the microenvironment variables: neovasculature.

The majority of therapeutic radiopharmaceuticals in current medical practice are *small molecules*, in contrast to large molecules such as monoclonal antibodies (mAb). Since the pharmacokinetics of these two groups are significantly different, we decided to select a *small molecule*. Considering the availability of experimental data, clarity of biochemical processes, and relevance to tumor biology, [^18^F]-Fluorodeoxyglucose ([^18^F] FDG) was selected to represent small molecule kinetics, while our intention is to extend and apply this framework to theranostics in future efforts.

Several decades ago, biochemical studies in tissue cultures demonstrated that metabolic abnormalities, especially malignant tumors, show high rates of glucose uptake and glycolysis^25^. [^18^F]-Fluorodeoxyglucose ([^18^F] FDG) PET allows the use of these metabolic abnormalities of tumors for clinical diagnosis^26,27^. The FDG uptake into tissue reflects the transport and phosphorylation of glucose by viable cells, where FDG is transported across the cell membrane and subsequently is phosphorylated by hexokinase. In contrast to glucose, FDG-6-phosphate cannot be further metabolized; moreover, because FDG-6-phosphate is a highly polar molecule, it cannot diffuse out of the cell and remains intracellular. Therefore, due to this trapping mechanism, the concentration of FDG is steadily increasing in metabolically active cells, and as a result, the contrast between the tumor and normal tissue increases.

The angiogenesis process, formation of new blood vessels from pre-existing blood vasculature, is a crucial component for supporting tumor growth by providing a source of nutrients and oxygen^28,29^. When the volume of the primary tumor exceeds a threshold ($\sim$ 1 mm^3^)^30^, its central cells become deficient in oxygen (hypoxic state); in response, the tumor and the cells surrounding secrete a variety of chemotactic and morphogenic tumor angiogenesis factors (TAFs) such as vascular endothelial growth factor (VEGF)^31^. The binding of TAFs to its receptor on the ECs of the existing vessel wall leads to the differentiation of ECs and the formation of tip endothelial cells (tECs) with increased motility. The tECs migrate toward the tumor in response to the TAF gradient (i.e., chemotaxis) and adhesion gradient to fibronectin (i.e., haptotaxis)^32^. Extend of tECs from the existing vessel wall, create new blood vessel sprouts in the initial stages of angiogenesis^33^. Transverse movement of the tECs leads migrating vessels to meet and forms connections to create a complex network of immature microvessels that supports blood flow into the tumor. Therefore, the process of tumor-induced sprouting angiogenesis is governed by the coordinated cell dynamics, which involves initial sprouting, branching, anastomosis, and vessel remodeling^34^.

Current mathematical simulation models of tumor angiogenesis can be classified into three categories: continuous, discrete, and hybrid models. The continuous approach assumes the vascular cells as bulk and continuous aggregates without considering the cells alone^35–38^. Although this model provides good qualitative results, it can not model the morphological details of the vascular network structure. The discrete models assume a set of defined rules and building-blocks structure very close to reality to simulate EC migration and the creation of new vessels^39–41^. Hybrid models consist of a combination of continuous and discrete models that trace the EC pathways using variable motion probabilities^42–45^.

In this study, we present the design and implementation of a comprehensive spatio-temporal multi-scale framework to collectively model the radiopharmaceutical distribution for different tumor sizes and microvessel densities (MVD). Our model includes subcellular, cellular, and tissue level size scales. The subcellular scale consists of biochemical agents fibronectin and tumor angiogenesis factor. The cellular scale includes the movement of endothelial cells and uptake of radiopharmaceuticals into the tumoral and normal cells. At the tissue scale, blood vessel growth is implemented by a hybrid method for the movement of tECs in two-dimensional interstitial space. We also model dynamic simulation of blood flow/pressure, which is determined by mechanobiological and biochemical signals from wall shear stress, pressure, and metabolic stimuli with accurate hemorheology and hemodynamics at the tissue level. In addition, the interstitial fluid flow that influences the distribution and transport of radiopharmaceuticals is also calculated. Subsequently, radiopharmaceuticals transport in different scales is analyzed using SDM equations.

## Methods

**Figure 1:**
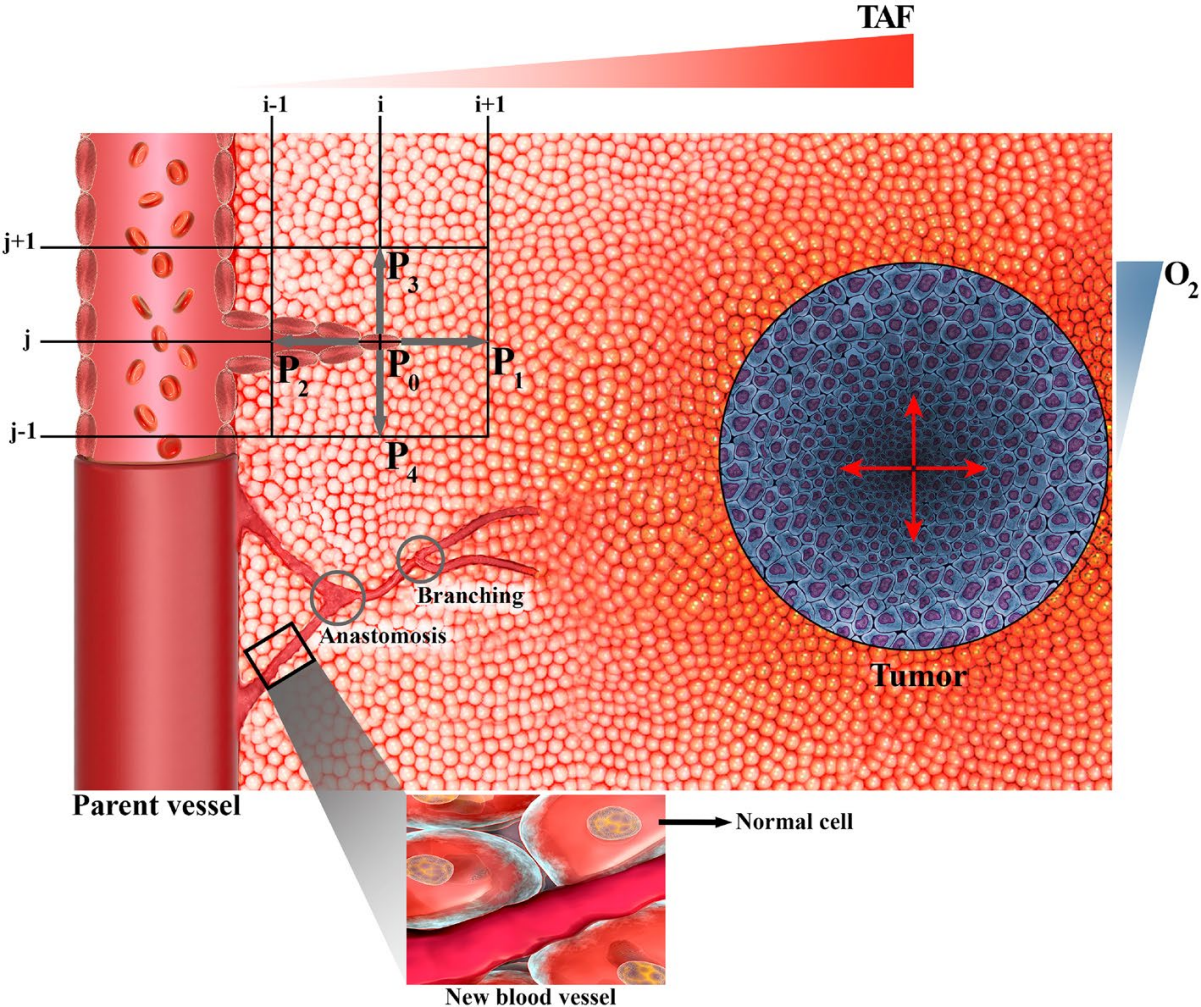
Schematic illustration of the capillary growth process based on the movement of single tip endothelial cells (tECs) in response to the tumor angiogenesis factors (TAFs) gradient within the computational domain. The migration of individual cell at each node of computational lattice (finite difference discretization) is determined in five states by ${P}_{i}$ coefficients, which include the probability of the cell being stationary (${P}_{0}$), or moving right (${P}_{1}$), left (${P}_{2}$), up (${P}_{3}$), or down (${P}_{4}$). Anastomosis and branching occur during the angiogenesis process. It should be mentioned that Microsoft Office PowerPoint 365 was used to create this figure.

For our modeling, we assume a semi-realistic micro-scale architecture of vasculature, interstitium, and tumor, as schematically depicted in Fig. 1. In this structural schema, various biochemical (e.g., TAF and O_2_) have spatially heterogeneous concentrations, and their gradients are highlighted at the border. In the following section, first, we present a mathematical model of angiogenesis and dynamic simulation of blood flow. Subsequently, spatiotemporal modeling of radiopharmaceutical transport in different scales will be described. Computational implementation will be discussed at the end (including geometry, computational domain, boundary conditions, and other simulation parameters).

### Mathematical model of angiogenesis

The present angiogenesis model is inspired by the tumor-induced angiogenesis model initially proposed by Anderson and Chaplin^43,45^. The mathematical model predicts capillary formation by tracking the motion of ECs at the tip of the capillary sprout, ultimately forming a vascular network. The details of the rules have been determined in the previous publication^43,45^. In summary, three main mechanisms for the motion of ECs are considered in this model: random motility, chemotaxis, and haptotaxis. Each motion component is the function of the motion stimulus gradient.

Random motility is modeled like diffusion of mass due to the concentration gradient. Chemotaxis is related to the TAF concentration gradient. TAF diffuses through the interstitium and activates the ECs. The distribution of TAF has its maximum value near the tumor zone and decreases to its minimum near the parent vessel (Fig. S1). Haptotaxis is the motion of cells due to the adhesion gradient in the environment. Cells move in search of stronger bonds in the interstitium, which is expressed in relation to the fibronectin concentration gradient in the environment. The complete system of equations describing the interactions between EC density, $n$, fibronectin, $f$ and TAF, $c_{TAF}$, are respectively written in Eqs. (1–3) as^43,45^:

1 $$\frac{\partial n}{{\partial t}} = \underbrace {{D_{n} \nabla^{2} n}}_{random\ motility} - \underbrace {{\nabla \cdot \left[ {\chi \left( {1 + \alpha c_{TAF} } \right)n\nabla c_{TAF} + \phi n\nabla f} \right]}}_{chemotaxis\ and\ haptotaxis}$$

2 $$\frac{\partial f}{{\partial t}} = \underbrace {\beta n}_{production} - \underbrace {\gamma nf}_{uptake}$$

3 $$\frac{{\partial c_{TAF} }}{\partial t} = \underbrace {{ - \eta nc_{TAF} }}_{uptake}$$

${D}_{n}$ is the random motility coefficient of EC, $\chi$ is the chemotaxis coefficient, $\alpha$ is constant of chemotaxis coefficient, $\phi$ is haptotaxis coefficient, $\beta$ and $\gamma$ are production and uptake coefficient of fibronectin, respectively, and $\eta$ is uptake coefficient of TAF by ECs. Implementation of the angiogenesis method to calculate the movement probabilities for each endothelial cell derived from these equations is presented in Supplementary Material (Eqs. (S1-S8)).

### Hemodynamics and interstitial fluid flow

For the fluid dynamics simulation model, we consider flow in three regions: intravascular blood flow, transvascular fluid flow, and interstitial fluid flow. To study the incompressible flow through the microvascular network, the flow rate in each vessel is calculated by applying mass conservation at each network junction. The continuity equation for intravascular blood flow at an interconnecting point, like c, in the network is written as Eq. (4) (Fig. S3). In this equation, the index $k$ refers to adjacent nodes, ${Q}_{c}^{k}$ is the net blood flow rate for each capillary calculated as the difference between the intravascular blood flow rate, ${Q}_{b.c}^{k}$, and the transvascular fluid flow rate, ${Q}_{t.c}^{k}$. The Womersley flow number for blood flow in the capillary network is very low due to the very small diameter of the newly created vessels. Hence, the pulsatile effects of the cardiac cycle can be ignored. Thus, Hagen-Poissville's law can be applied for intravascular blood flow as an exact solution to the Navier–Stokes fluid dynamics equation (Eq. 5)^41^. Transvascular fluid flow rate is calculated by Starling's law, which indicates the role of oncotic and hydrostatic pressures in fluid movement across capillary membranes (Eq. 6)^41^.

4 $$\mathop \sum \limits_{k = 1}^{N} Q_{c}^{k} \beta_{c}^{k} = 0,\quad Q_{c}^{k} = Q_{b,c}^{k} - Q_{t,c}^{k}$$

5 $${Q}_{b,c}^{k}=\frac{\pi }{128}\frac{\Delta {P}_{b}{d}^{4}}{l \mu (d,H)}$$

6 $${Q}_{t,c}^{k}=\pi dl{L}_{p}[{\overline{P} }_{b}-{\overline{P} }_{i}-({\pi }_{b}-{\pi }_{i})\sigma ]$$

In Eq. (4), $N$ is the number of peripheral vessel lattice nodes adjacent to the central vessel node. In the 2-D simulation for a fully connected network, $N$ is 4 and ${\beta }_{c}^{k}$ is a positive integer '0' or '1', which describes whether nodes k and c are connected (${\beta }_{c}^{k}$ = 1) or not (${\beta }_{c}^{k}$= 0). In Eq. (5), ${P}_{b}$ is intravascular pressure (IVP), H is hematocrit, and $d$ and $l$ are the diameter and length of the new vessel, respectively. In Eq. (6), ${L}_{p}$ is the hydraulic conductivity of the microvascular wall, ${\overline{P} }_{b}$ is the average IVP in each element, ${\overline{P} }_{i}$ is the average interstitial fluid pressure (IFP) outside of the vascular element, ${\pi }_{b}$ is the osmotic pressure of the intravascular plasma, ${\pi }_{i}$ is the osmotic pressure of the interstitial fluid, and $\sigma$ is the average osmotic reflection coefficient for plasma proteins.

IVP is calculated by applying Poiseuille’s equation for the flow in the vessels. In contrast, interstitial pressure for the peripheral tissue of a microvascular network is calculated by solving the governing equation for fluid flow through a porous medium. The mass balance equation for a steady-state incompressible fluid is modified by adding source and sink terms for biological tissues (Eq. 7). Blood vessels are fluid source terms, and lymphatic vessels are sink terms, which are respectively shown in Eqs. (8) and (9), according to Starling's law. Moreover, Darcy’s experimental observations show that the interstitial fluid velocity (IFV) in porous media is proportional to the pressure gradient (Eq. 10).

7 $$\nabla \cdot {v}_{i}={\phi }_{b}-{\phi }_{L}$$

8 $${\phi }_{b}=\frac{{L}_{P}S}{V}\left({\overline{P} }_{b}-{\overline{P} }_{i}-\sigma \left({\pi }_{b}-{\pi }_{i}\right)\right)$$

9 $${\phi }_{L}=\frac{{L}_{PL}{S}_{L}}{V}({\overline{P} }_{i}-{P}_{L})$$

10 $${v}_{i}=-K\nabla {P}_{i}$$

${v}_{i}$ is the IFV, ${\phi }_{b}$ is the rate of fluid flow per unit volume from blood vessels into the interstitial space, ${\phi }_{L}$ is the rate of fluid flow per unit volume from the interstitial space into lymph vessels, $S/V$ is the surface area of the microvascular per unit volume for mass transport in the interstitium, ${P}_{L}$ the hydrostatic pressure of the lymphatic, and $K$ is the hydraulic conductivity of the interstitium.

By combining Eqs. (7) and (10), we can derive the Poisson-Laplace’s equation to calculate IFP in both normal and tumor tissues (Eq. 11).

11 $$\begin{gathered} - \nabla^{2} P_{i} = \left\{ {\begin{array}{*{20}c} {\left\{ {\begin{array}{*{20}c} {M - N } \\ {M } \\ \end{array} } \right.} \\ {\left\{ {\begin{array}{*{20}c} { - N } \\ {0 } \\ \end{array} } \right.} \\ \end{array} } \right. \begin{array}{*{20}c} {\begin{array}{*{20}c} {Normal\ tissue} \\ {Tumor\ tissue} \\ \end{array} } & {For\ existence\ blood\ source} \\ {\begin{array}{*{20}c} {Normal\ tissue} \\ {Tumor\ tissue} \\ \end{array} } & {Otherwise } \\ \end{array} \hfill \\ M = \frac{{L_{P} S}}{KV}\left( {\overline{P}_{b} - \overline{P}_{i} - \left( {\pi_{b} - \pi_{i} } \right)\sigma } \right) \hfill \\ N = \frac{{L_{PL} S_{L} }}{KV}\left( {\overline{P}_{i} - P_{L} } \right) \hfill \\ \end{gathered}$$

### Hemorheology

Blood has significant non-Newtonian properties in low Reynolds numbers. We assume that blood is a suspension of non-Newtonian red blood cells within a Newtonian plasma fluid^46–48^. Pries et al.^49^ presented an empirical relation for the dynamic blood viscosity in capillaries, shown in Eq. (12). In Eq. (12), ${\mu }_{blood}$, ${\mu }_{plasma}$ and ${\mu }_{rel}$ are the dynamic viscosity of blood and plasma and apparent viscosity of blood, respectively. The apparent blood viscosity is defined as a function of vessel diameter and hematocrit by Eq. (13). ${\mu }_{0.45}$ is the relative apparent blood viscosity for a fixed hematocrit of 0.45, which is defined in Eq. (14) as a function of vessel diameter, and C is a function that describes the shape of viscosity dependency on hematocrit, which is defined in Eq. (15). In Eqs. (13–15), the unit of $d$ is the $\mu m$.

12 $${\mu }_{blood}={\mu }_{rel}\cdot {\mu }_{plasma}$$

13 $${\mu }_{rel}=\left[1+\left({\mu }_{0.45}-1\right)\frac{{\left(1-H\right)}^{C}-1}{{\left(1-0.45\right)}^{C}-1}{\left(\frac{d}{d-1.1}\right)}^{2}\right]{\left(\frac{d}{d-1.1}\right)}^{2}$$

14 $${\mu }_{0.45}=3.2+6\mathrm{\exp}\left(-0.085d\right)-2.44\mathrm{exp}\left(-0.06{d}^{0.645}\right)$$

15 $$C=\left(0.8+\mathrm{exp}\left(-0.075d\right)\right)\left[\frac{1}{1+{10}^{-11}{d}^{12}}-1\right]+\frac{1}{1+{10}^{-11}{d}^{12}}$$

Hematocrit distribution has a significant role in simulating the hemodynamic characteristic of the microvascular network, which depends on blood flow characteristics such as velocity. In general, the hematocrit distribution at vessel bifurcations can change depending on the flow velocity in each branch. In other words, if the velocity ratio of the two branches exceeds the threshold value, ${U}_{cr}$, all hematocrit enter the faster branch at bifurcations^50^. According to this, the relation between the hematocrit of the parent vessel, ${H}_{i}$, and the branches, ${H}_{1}$ and ${H}_{2}$, are written as Eqs. (16–18), respectively, based on the velocity ratio of the two branches, ${U}_{1}/{U}_{2}$. $\lambda$ is a phenomenological parameter that accounts for the strength of the non-symmetry of the hematocrit distribution at bifurcations^50^.

16 $$H_{i} = H_{1} + H_{2} ,\quad U_{1} > U_{2}$$

17 $$if \frac{{U}_{1}}{{U}_{2}}>{U}_{cr} \to \left\{\begin{array}{c}{H}_{1}={H}_{i}\\ {H}_{2}=0\end{array}\right.$$

18 $$if \frac{{U}_{1}}{{U}_{2}}<{U}_{cr} \to \frac{{H}_{1}}{{H}_{2}}=\lambda \frac{{U}_{1}}{{U}_{2}}$$

### Dynamic structure adaptation method

Capillaries are able to continuously adapt their diameter in response to physical and biochemical stimuli of the tissues that the capillaries supply^47,49^. The wall shear stress stimuli, ${S}_{wss}$, and the IVP created by blood flow,${S}_{p}$, and the biochemical stimuli such as the hematocrit-induced metabolic, ${S}_{m}$, lead to the remodeling of the vascular diameter, shown in Eqs. (19–21), respectively. ${\tau }_{w}$ is the wall shear stress in a capillary vessel defined by Eq. (22). Experimental observations show that when each vessel senses the shear stress of the wall, it adjusts its diameter to achieve a uniform level of stress. ${\tau }_{ref}$ is a small constant to avoid singular behavior at low wall shear stress rates. ${\tau }_{e}({P}_{b})$ is the wall shear stress resulting from the blood pressure, ${P}_{b}$, defined in Eq. (23). ${Q}_{b}$ is the rate of blood flow in the vessel, and ${Q}_{ref}$ is the maximum value of ${Q}_{b}$ within the network.

19 $${S}_{wss}={Log}_{10}({\tau }_{w}+{\tau }_{ref})$$

20 $${S}_{p}=-{Log}_{10}{\tau }_{e}({P}_{b})$$

21 $${S}_{m}={Log}_{10}\left(\frac{{Q}_{ref}}{{Q}_{b}H}+1\right)$$

22 $${\tau }_{w}=\frac{32{\mu }_{blood}Q}{\pi {d}^{3}}$$

23 $${\tau }_{e}({P}_{b})=100-86\cdot \mathrm{exp}\left(-5000\cdot {\left({Log}_{10}\left({Log}_{10}{P}_{b}\right)\right)}^{5.4}\right)$$

Finally, the change of diameter for each vessel in the network in proportion to the total signal and time step is considered in Eq. (24). ${k}_{p}$ is the adaptive response sensitivity of the vessel diameter to changes in IVP, ${k}_{m}$ is the adaptive response sensitivity of the vessel diameter to changes in metabolic state, and ${k}_{s}$ is the shrinking tendency of the vessel in the absence of positive growth stimuli.

24 $$\frac{d(d)}{dt}=({S}_{wss}+{{k}_{p}S}_{p}+{{k}_{m}S}_{m}-{k}_{s})$$

### Spatiotemporal distribution of FDG

In PET, images are a composite of different superimposed signals. A signal, for example, may describe the amount of the phosphorylated radiopharmaceutical. In order to separate the desired component of the signal, a mathematical model is required that relates the dynamics of all radiopharmaceutical states to the resulting PET image. Mathematical kinetic models are used to analyze the time sequences of PET images, in which each of the radiopharmaceutical states is known as a compartment. Each compartment is determined by the concentration of the radiopharmaceutical inside it as a function of time. These concentrations are related through a set of ODEs, which express the balance between the input and output of each compartment. In compartmental modeling, it is assumed that there are no spatial concentration gradients within the sampled area (e.g., a voxel). The directly measured radiopharmaceutical concentration in blood as a function of time acts as a model input function. The coefficients of the differential equations in the model are considered as constants that reflect the inherent kinetic properties of the specific radiopharmaceutical molecule in the system. By formally comparing the model output to the experimentally obtained PET data, the values of these kinetic parameters can be estimated, and information about the delivery process can be extracted^51^. The 4-compartment 5-rate-constant (5 K) model distinguishes the kinetic steps of [^18^F]FDG delivery to the extracellular matrix (ECM), its transport from the extracellular to the intracellular space, and its intracellular phosphorylation^16^. The 5 K model is described by ODEs, shown in Eqs. (S9–S11) at the Supplementary Material, and can be viewed as an extension of the classic model by Sokoloff et al.^15^. In fact, a value of the 5 K model is in its explicit accounting of an extracellular compartment.${K}_{1}$ and ${K}_{2}$ are exchange rate parameters between plasma and extracellular space, ${K}_{3}$ and ${K}_{4}$ are transport rate parameters into and out of the cell, and ${K}_{5}$ is the phosphorylation rate.

**Figure 2:**
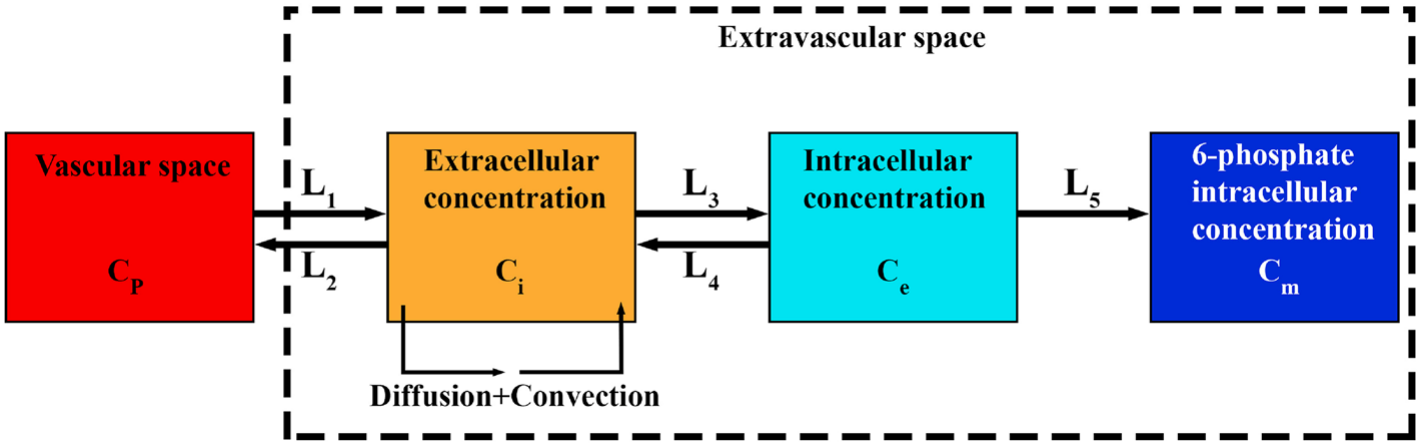
Four-compartment 5-rate-constant model for spatiotemporal modeling of [^18^F]FDG distribution.

As mentioned before, in contrast to classic compartment model, the SDM model uses PDEs that enables the evaluation of radiopharmaceutical distribution in both space and time scales, hence distribution in space is not modeled as independent from time. Fig. 2 describes the SDM model. From the arterial blood, the radiopharmaceutical passes into the second compartment, known as the extracellular compartment, where it is considered that the radiopharmaceutical is redistributed in both time and space. The third and fourth compartments are the region of intracellular radiopharmaceutical. Movement between the plasma, extracellular, and intracellular spaces is governed through five parameters: ${L}_{1}$, ${L}_{2}$, ${L}_{3}$, ${L}_{4}$, and ${L}_{5}$, similar to the rate constants ${K}_{1}$, ${K}_{2}$, ${K}_{3}$, ${K}_{4}$, and ${K}_{5}$ seen in conventional compartmental models. The general form of the SDM equations in tissue is shown by Eqs. (25–27) as^52^:

25 $$\frac{\partial {C}_{i}}{\partial t}={D}_{eff}{\nabla }^{2}{C}_{i}-{v}_{i}\cdot \nabla \left({C}_{i}\right)+{L}_{1}{C}_{P}-({L}_{2}+{L}_{3}){C}_{i}+{L}_{4}{C}_{e}$$

26 $$\frac{\partial {C}_{e}}{\partial t}={L}_{3}{C}_{i}-{(L}_{4}+{L}_{5}){C}_{e}$$

27 $$\frac{\partial {C}_{m}}{\partial t}={L}_{5}{C}_{e}$$

28 $${L}_{1}=\frac{{L}_{P}S}{V}\left({P}_{b}-{P}_{i}-\left({\pi }_{b}-{\pi }_{i}\right)\sigma \right)(1-{\sigma }_{f})+\frac{{P}_{m}S}{V}\frac{Pe}{{e}^{Pe}-1}$$

29 $${L}_{2}=\left(\frac{{P}_{m}S}{V}\frac{Pe}{{e}^{Pe}-1}\right)+{\phi }_{L}$$

30 $$Pe = \frac{{\phi_{b} \left( {1 - \sigma_{f} } \right)}}{{P_{m} \frac{S}{V}}}$$

where ${C}_{i}$ is extracellular concentration of [^18^F]FDG, ${C}_{e}$ is [^18^F]FDG intracellular concentration, and ${C}_{m}$ is [^18^F]FDG 6-phosphate intracellular concentration. Furthermore, ${D}_{eff}$ is the effective spatially-invariant diffusion coefficient, ${v}_{i}$ is the IFV vector,${\sigma }_{f}$ is the filtration reflection coefficient, ${P}_{m}$ is the vascular permeability coefficient, and $Pe$ represents the Peclet number, indicating the ratio of convection transport rate to diffusion transport rate across the vessel wall to interstitium. The first and second terms in the right-hand side of Eq. (25) determine the diffusion transport and convection transport of radiopharmaceuticals within the interstitium, respectively.

### Computational implementation

#### Computational geometry and simulation cases

**Figure 3:**
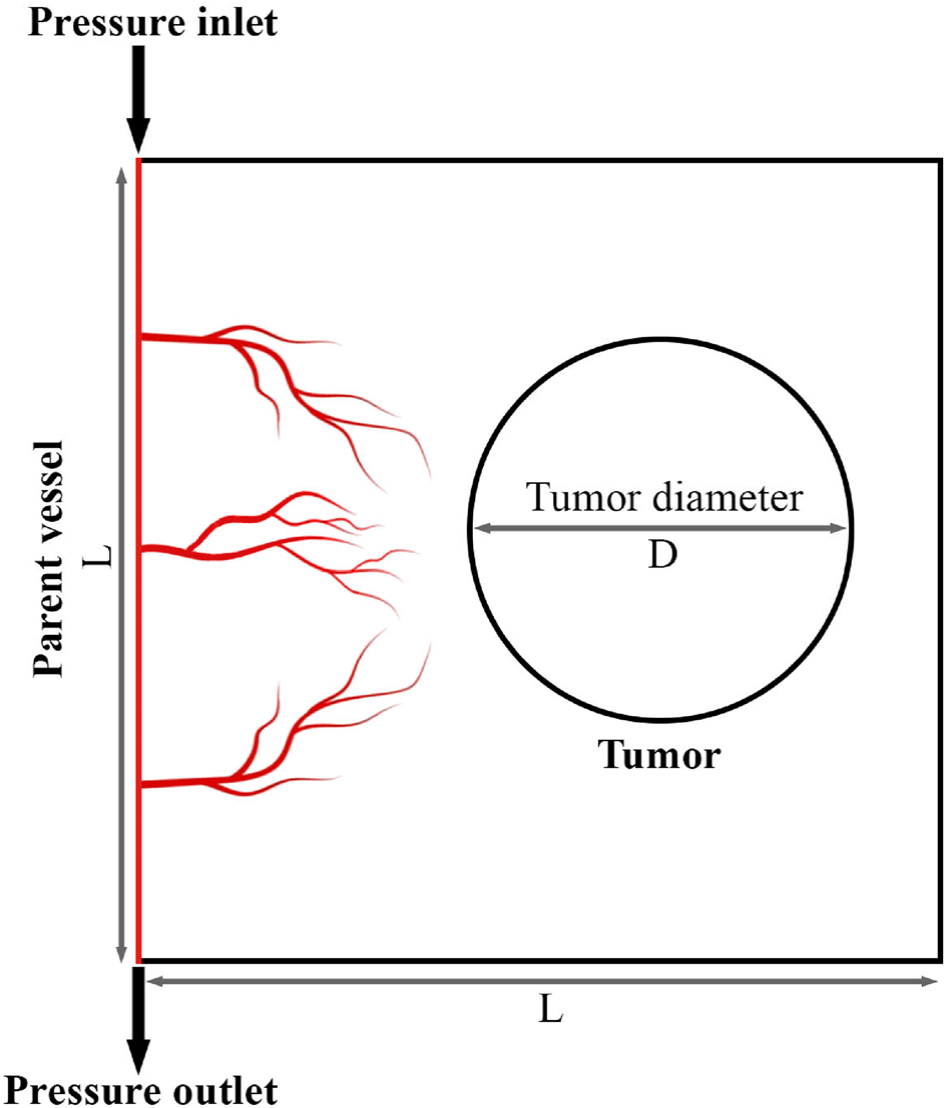
A schematic of the computational domain employed in the current study. The vascular network consists of a parent vessel located on the left side of the domain. It should be mentioned that Microsoft Office PowerPoint 365 was used to create this figure.

To reduce computational cost and simplify the calculations, we selected a $L\times L$ square as the computational domain representing TME and its surrounding normal tissues, as demonstrated in Fig. 3. The “D” in this figure shows the tumor size and is non-dimensionalized by the domain length ($\frac{D}{L} = 0.2$). A parent vessel is located the left edge of the domain where the vascular network starts to grow. To investigate the effect of tumor size, three different tumor sizes including *D*, $2\times D$, and $3\times D$ are considered. On the other hand, to examine the impact of MVD within the TME, two different states are considered for initiating angiogenesis from the parent vessel, including network with 3 initial sprouts and network with 5 initial sprouts, at the same distance from each other. The values of the model parameters used in this study are listed in Table S2 of the Supplementary Material.

#### Solution strategy

**Figure 4:**
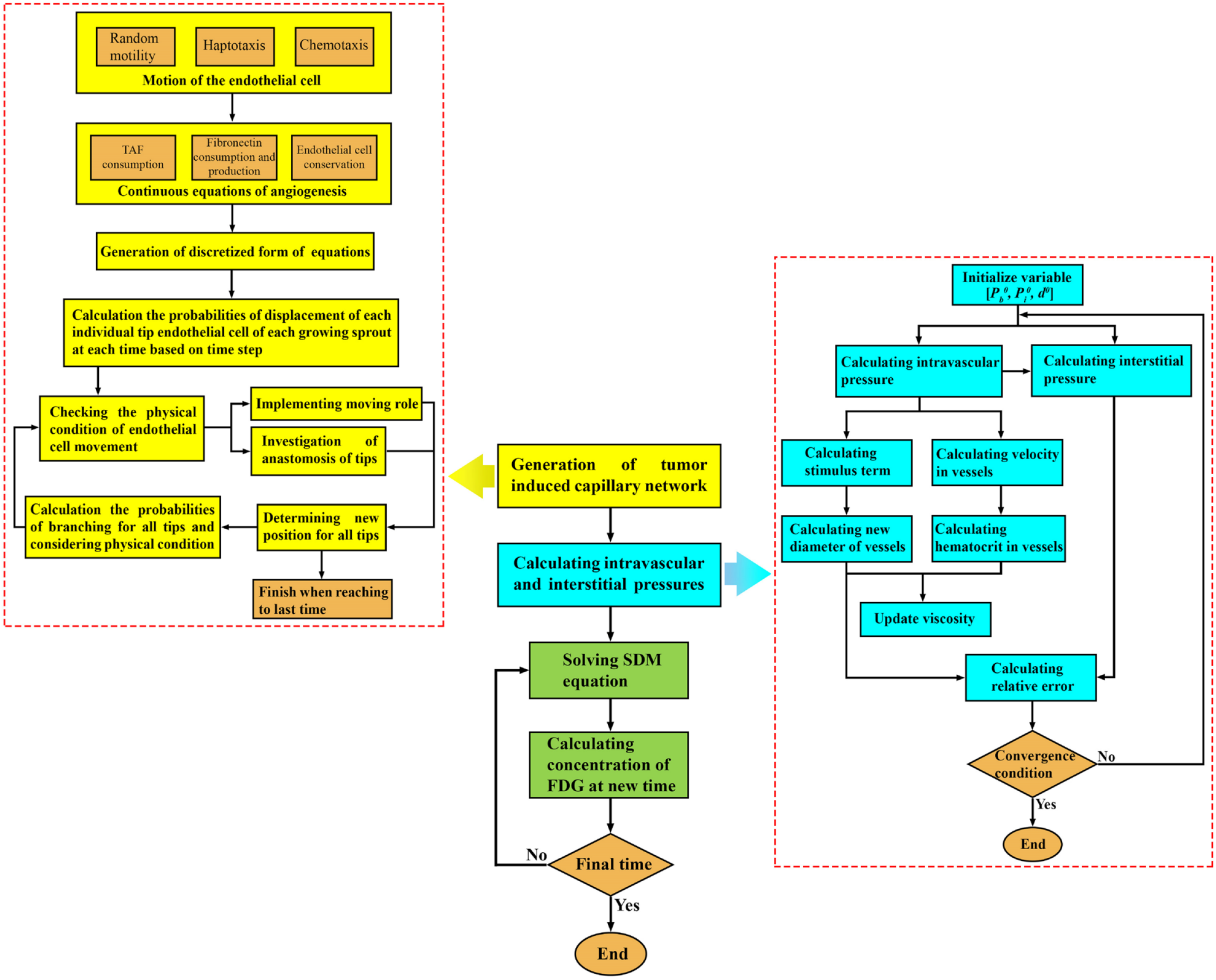
A step-by-step flowchart of the present multi-scale model (generation of the capillary network corresponds to one time step).

In this study, a two-dimensional hybrid sprouting angiogenesis method is applied to produce the microvascular network. The governing equations are discretized by a finite difference scheme to a set of algebraic equations. The solution of the discretized equations is performed in an iterative process by MATLAB. The calculation of blood flow in capillaries includes a set of non-linear equations. Hence, an iterative method is applied to solve the blood flow equations in the capillary network by MATLAB (160,000 elements). Subsequently, the momentum and mass equations in the microvascular network and interstitial space are solved. The resulting IFP and IFV values are used for solving the SDM equations to obtain the distribution of different concentrations (28,082 elements). The initial and boundary conditions of this study are mentioned in Supplementary Material (Table S1). A step-by-step flowchart of the present multi-scale model is reflected in Fig. 4. The Darcy, and SDM equations are solved by the commercial finite element software COMSOL Multiphysics version 5.5a (COMSOL Inc., Stockholm, Sweden). In addition, the residual square errors are set to 4 orders of magnitudes and the time of analysis for the FDG distribution is considered 1 h. All simulations described in this manuscript were performed on an Intel Core i5-8250U, 1.8 GHz CPU and RAM 8 GB computer.

## Results and discussion

### Morphology of the angiogenesis network

**Figure 5:**
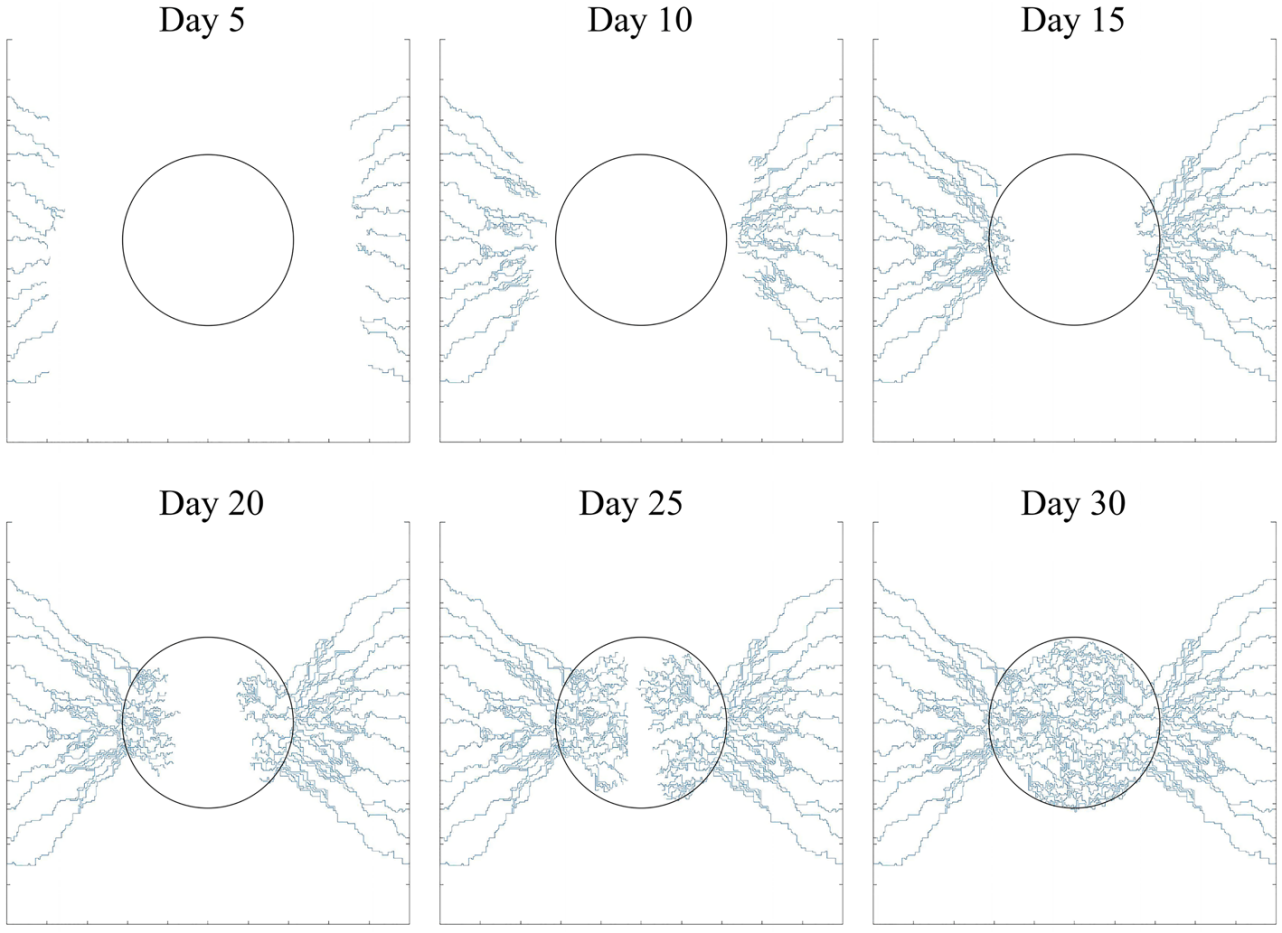
Different stages of microvascular network formation during angiogenesis at days 5, 10, 15, 20, 25, and 30.

To analyze the different stages of tumor angiogenesis, a sample vascular network induced by two parent vessels on the left and right sides of the domain has been generated over 30 days (Fig. 5). At 5 days, the TAF forms a gradient in the domain that reaches the parent vessels and causes sprouting angiogenesis to form new vessels. Between days 5–15, the generated vessels are elongate, branch, deform and spread toward the tumor. After day 15, new vessels have been extended within the TME to reach together and form vascular loops.

**Figure 6:**
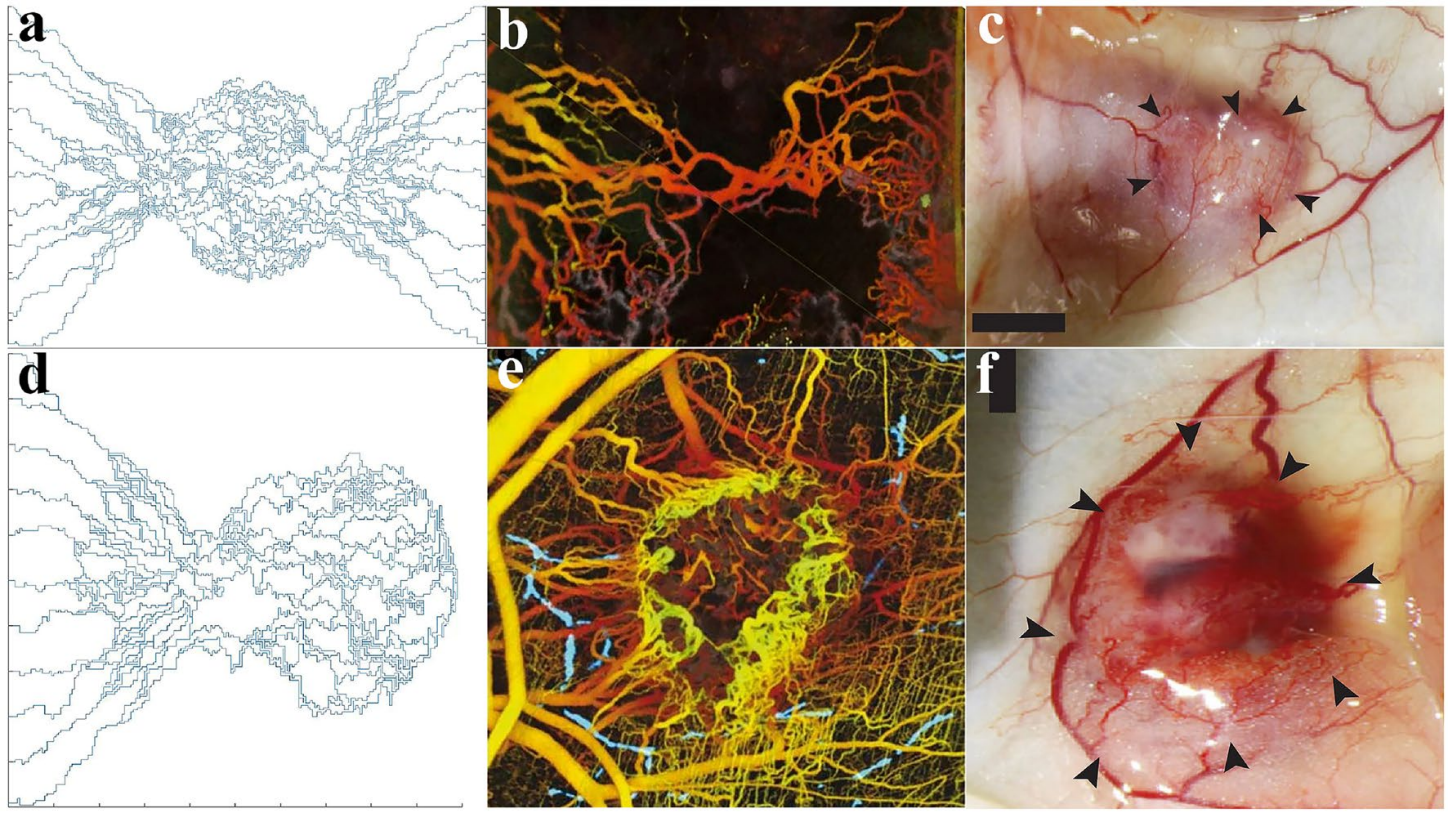
Biological validation of the angiogenesis modeling. (**a**) Our simulations show that the branching of new vessels occurs near the tumor boundary and inside the tumor; in vivo observations of tumor angiogenesis by (**b**) Vakoc et al.^53^ and (**c**) Roudnicky et al.^54^. (**d**) The sprouting angiogenesis induced by one parent vessel form vascular loops; and in vivo observations of tumor angiogenesis by (**e**) Vakoc et al.^53^ and (**f**) Roudnicky et al.^54^. It should be mentioned that (**b**) and (**e**) are extracted from Vakoc et al.^53^ with permissions. Additionally, (**c**) and (**f**) are taken from Roudnicky et al.^54^ with permissions.

A qualitative comparison between the results of this study and biological observations of tumor angiogenesis morphology^53,54^ is shown in Fig. 6. In agreement with in vivo observations, the simulations show that the branching of new vessels increases near the tumor boundary as well as within the tumor where the TAF concentration is high (Fig. 6a–c), because TAF induces new tECs and create new branches. As mentioned, to reduce computational cost and simplify the calculations, the angiogenesis model caused by one parent vessel has been considered in this study (Fig. 6d). This assumption is biologically validated and confirmed by Fig. 6e,f.

In the calculations, given the sake of investigating the dependency of FDG radiopharmaceutical activity on the tumor size as well as MVD within the tumor, six capillary networks—including three different tumor sizes (*D*, $2\times D$, and $3\times D$) in two different states of initiating angiogenesis (with 3 initial sprouts and 5 initial sprouts)—have been generated and examined in detail, as demonstrated in Fig. S2 of Supplementary Material. It should be noted that in a realistic capillary network, the maximum distance between the tumor cells and the closest microvessels is considered to be about 100 μm^55^. This is not considered in our microvascular networks, to more clearly investigate the effect of MVD and the distribution of solute within the tumor. However, this assumption satisfies the mechanisms of extravasation from microvessels and transport in interstitium. In fact, MVD plays a potential role as prognostic biomarker for different cancers including breast^56,57^ and has been shown as elevated in higher-grade tumor tissues compared to lower-grade ones^58^. Meanwhile, different sizes of tumors can imply different stages of tumor growth^59^.

### Pressure and velocity distribution

The computational domain includes two different tumor and normal tissues. The capillary network is used to simulate IVP and IFP. The various mechanisms of transport, namely diffusion and convention from vessel to tissue and within a tissue, are elaborately calculated across the computational domain. The diffusion terms are related to concentration gradient and the convection terms are related to interstitial flow. The distribution of IVP in networks with adaptable capillaries are shown in Fig. S4 of Supplementary Material. It can be seen that the inlet sides of the parent vessel have the maximum values of IVP and it decreases towards the outlet to the minimum values. The IVP is also adjusted based on the pressure drop of the parent vessel, which causes blood flow. The obtained results are in very good agreement with IVPs obtained by Soltani and Chen^41^ and Stylianopoulos et al.^60^.

**Figure 7:**
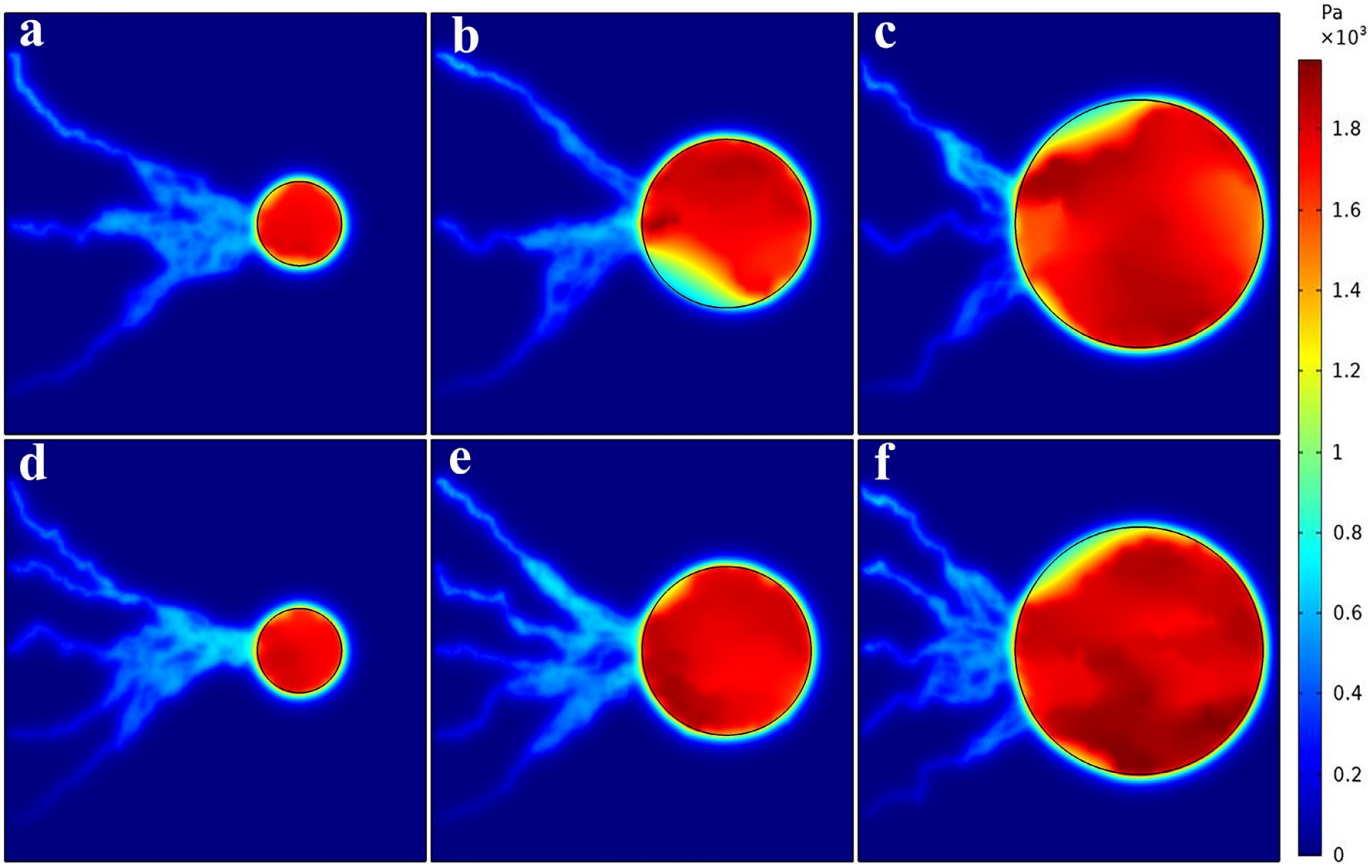
Interstitial fluid pressure distribution for six considered networks.

The distribution of IFP of tumor and normal tissues for six considered networks are shown in Fig. 7. The mean spatial value of tumor IFP for the networks 1–6 is 1525.7 Pa, 1585.5 Pa, 1698.3 Pa, 1570.2 Pa, 1700.7 Pa and 1868.2 Pa, respectively. The results are good compatible with the numerical studies of Soltani et al.^61^, Souri et al.^62^, Al-Zu’bi and Mohan^63^ as well as the experimental study of Butcher et al.^64^, which demonstrated the range of 586–4200 Pa for IFP in the tumor. Moreover, the IFP value in normal tissue is around 53.7–97.5 Pa, that agrees with the values of the experimental study^65,66^, which demonstrated the range of − 400 to 800 Pa for IFP in normal tissue. IFP has its greatest value in the tumor region because the lymphatic system is dysfunctional and the capillary network of the tumor has a higher leakage rate than that of normal tissue, leading to radiopharmaceutical accumulation in the tumor interstitium, and as a result, an elevated IFP.

IFP is known as an important barrier against efficient solute transport to tumor tissue. IFP is proportional to the MVD in tumor and normal tissues. Hence, the IFP value is higher in the area where the capillaries are closer together. In addition, tumor with higher MVD has greater IFP, according to the mean values of the measured IFP. As Fig. 7 illustrates, the value of IFP decreased rapidly at the tumor boundary because the transvascular flow is absorbed by the lymphatic vessels in the normal tissue region. Moreover, the heterogeneous capillary network, as the source term in the interstitial fluid flow equation, causes the heterogeneous distribution of IFP in tumor tissue.

**Figure 8:**
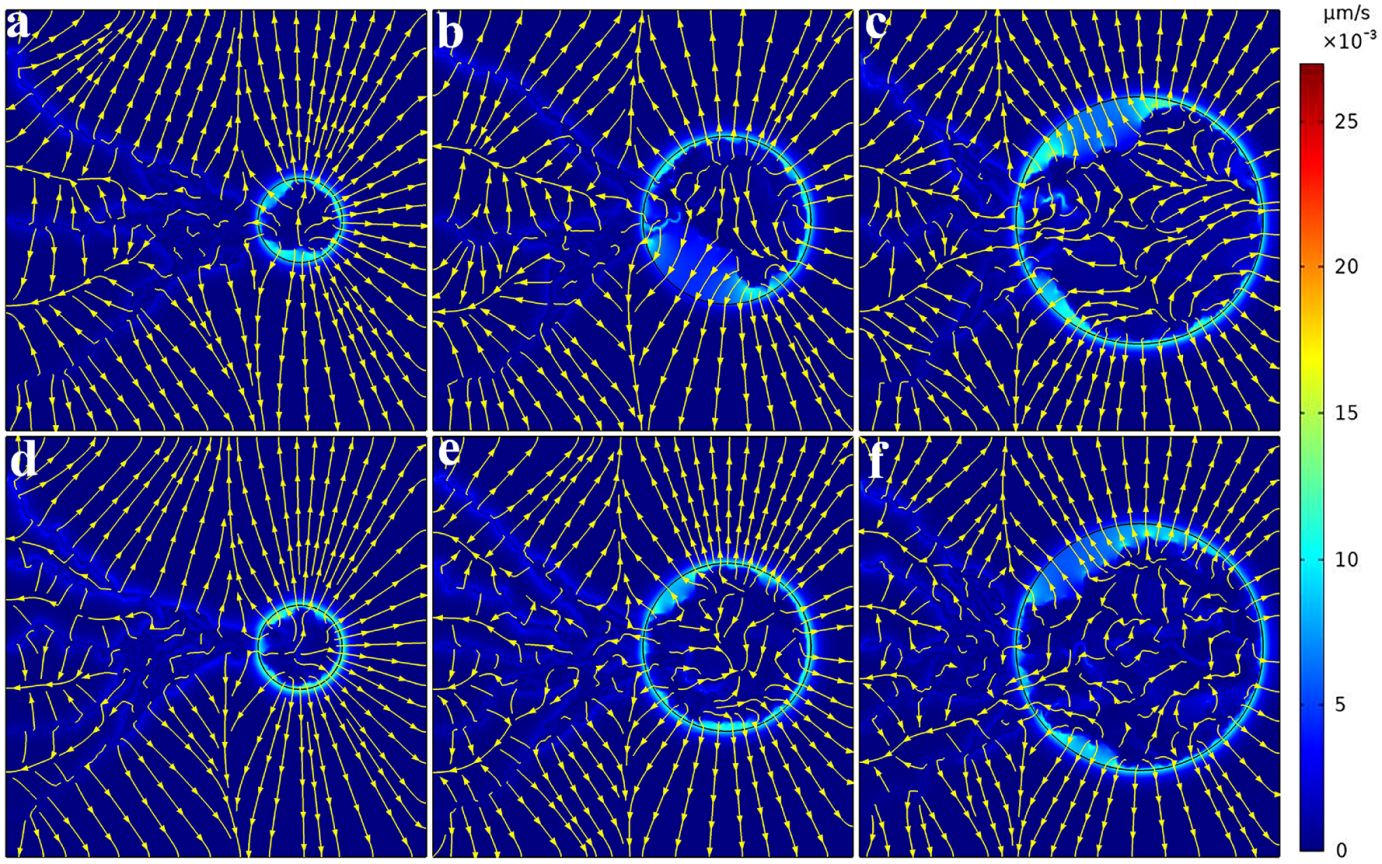
Interstitial fluid velocity distribution for six considered networks. Streamlines are given in each subfigure. IFV has very low values for both tumor and normal tissues. Maximum IFV occurs at the tumor boundary where there is a large IFP gradient. Fluid flow is directed from high pressure areas to low pressure areas.

Given a direct relationship between IFP and IFV based on Darcy's law, IFV distribution can be obtained in the whole tissue domain, as demonstrated in Fig. 8 for six considered networks. The velocity has very low values in most areas. The fluid flow direction is from high pressure area toward a low pressure area. Indeed, the maximum value of IFV occurs at the boundary between tumor and normal tissue where there is a large IFP gradient. IFV results are in the same order as the prediction of Jain et al.^38^, Soltani et al.^61^, Souri et al.^62^, Kashkooli et al.^67^, and experimental observation of Hompland et al.^68^.

In the following, the effect of five factors on flow simulation is examined:

#### Osmotic pressure

The transvascular flow, which is of crucial importance for the homeostasis of tissues, is determined by the hydrostatic and osmotic pressures in vessels and in the interstitium. In tumors, normal osmotic pressures necessary for the transit of solutes into and out of vessels along gradients are severely compromised owing to the local tumor microenvironment^69^. Hence, the hydrostatic pressure difference between intra- and extravascular spaces is the dominant term of transvascular flow^70^. Therefore, due to the minor role of osmotic pressure in the transvascular flow, it can be neglected^71^, although this assumption is far from the truth.

#### Hematocrit distribution

As can be seen from the equations, intravascular flow (${Q}_{b}$), dynamic blood viscosity (${\mu }_{blood}$), and metabolic stimuli for changing the diameter (${S}_{m}$) of each branch depend on the hematocrit in that branch. No change in hematocrit of the branches affects the mentioned cases. According to the researches on vascular dynamics, the hematocrit distribution in branches varies proportional to velocity.

#### Changing vessel diameter

If we call a network with change in diameter a “dynamic network” and a network with fixed diameter a “static network”, the dynamic network adapts its vessels diameter to reach enough blood flow in each vessel. Therefore, the intravascular pressure of the dynamic network is higher than the static network (Fig. S5). In addition, the dynamic network has a higher IFP because of the elevated intravascular blood pressure in the tumor, which increases the transvascular flow rate. The results of the blood pressure distribution for the static network led to an unrealistic distribution of solute concentrations.

#### Lymph pressure

Lymphatic vessels are absent or non-functional in tumors^72,73^. According to the equations, if the lymphatic term is considered in tumor, it will act as a sink term. Therefore, it removes both the drug and the fluid inside the tumor, which reduces the IFP and radiopharmaceutical accumulation. Moreover, if ${P}_{L}>0$, the difference between ${P}_{i}$ and ${P}_{L}$ becomes larger and the sink term effect decreases in normal tissue.

#### The removal of blind ends and void of flow vessels

Since a mathematical method is used to generate the network, all the generated branches may not be placed in a flow loop. These branches, which are generated due to the nature of the mathematical model, are not practical from a physical viewpoint. As stated by^74^ and applied in^41,75–77^, elimination of vessels which are void of flow has proven to be applicable in mathematical models. Therefore, the capillaries that have a flow rate of less than 1% of the network’s maximum flow rate can be eliminated. Bazmara et al. showed that in the absence of blood flow and shear stress, a loop cannot maintain its stability and collapses at the end^78^. Stephanou et al. investigated removing the vessels having flow less than 1% of the maximum capillary flow. It was observed that the flow distribution remains essentially unchanged when compared with the unmodified vasculature and a comparison of drug uptake in the two systems shows that maximum uptakes are also similar. However, the modified network initially delivers the drug more quickly: the treated vasculature has been optimized^79^.

### Distributions of FDG uptake

**Figure 9:**
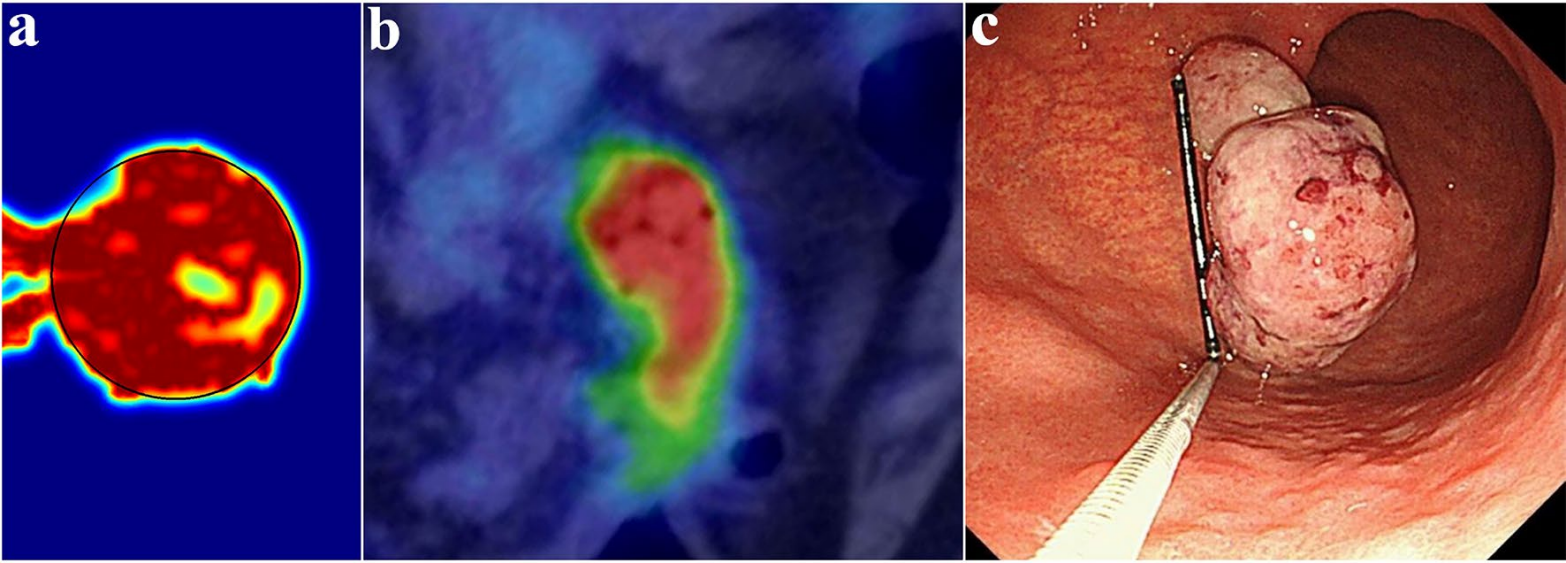
Qualitative validation of simulation results. (**a**) Synthetic PET image generated by the current mathematical model; (**b**) [^18^F]FDG PET imaging shows high uptake of [^18^F]FDG, suggesting the possibility of advanced gastric cancer^80^; (**c**) Upper gastrointestinal endoscopy image shows a large (3.5 cm) pedunculated polyp-shaped gastric cancer with prolapse into the duodenal bulb^80^. (Sections (**b**) and (**c**) are used with permission from Suzuki et al.^80^).

A qualitative comparison between the results of this study and the real FDG PET image of a large polyp-shaped gastric cancer for a 70-year-old man^80^ is shown in Fig. 9. In agreement with the real PET image, the simulations show high uptake of [^18^F]FDG in the tumor region. In addition, the created color gradient clearly demonstrates the difference between normal and tumor tissue. We emphasize that this comparison with experimental data is only for demonstration purpose and the simulated FDG image (Fig. 9a) does not use any clinical data.

**Figure 10:**
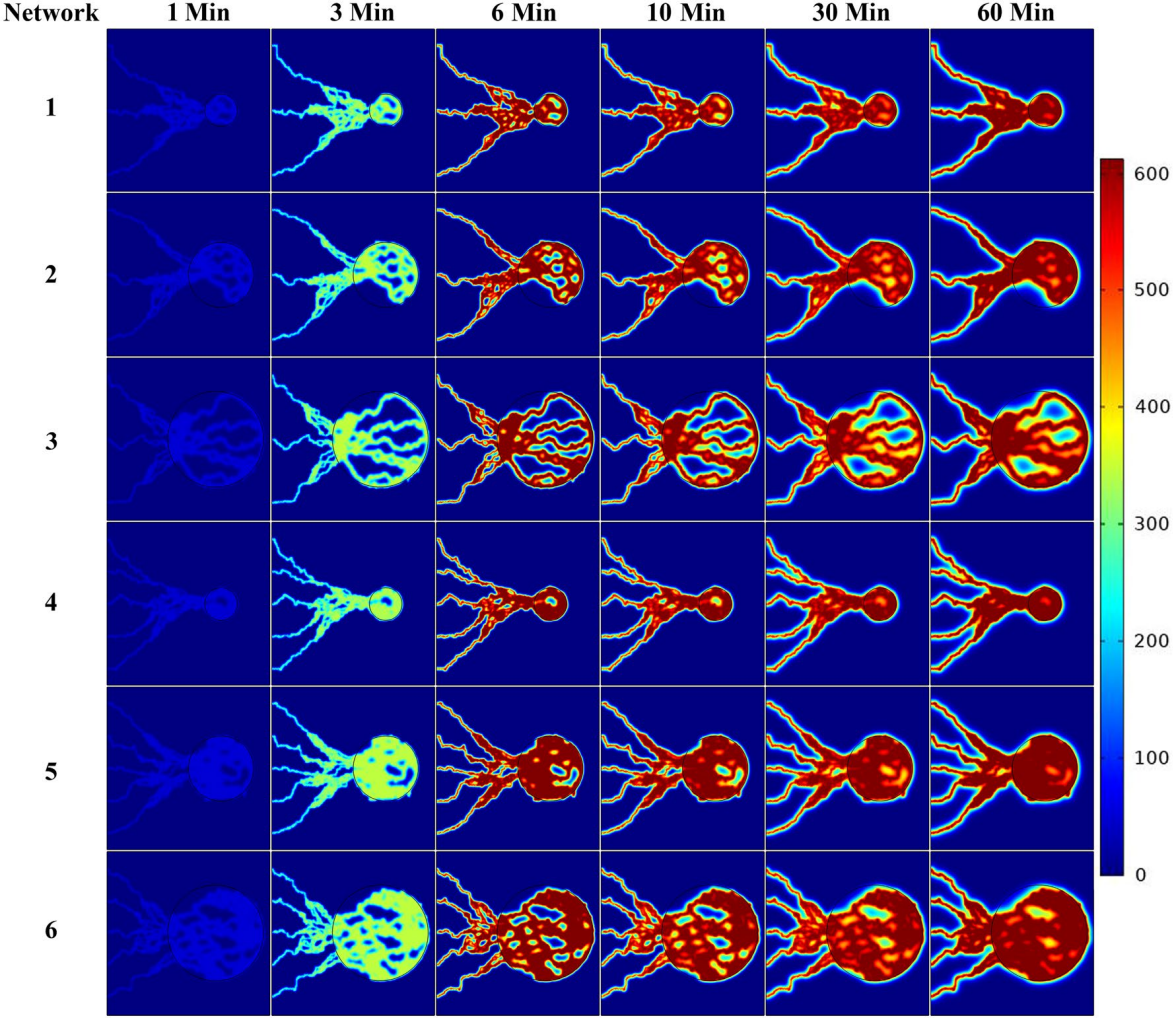
Spatiotemporal distribution of total FDG radiopharmaceutical concentration ($\frac{kBq}{ml}$) at 1, 3, 6, 10, 30 and, 60 min in six networks.

Since the plasma concentration of the radiopharmaceutical varies by time, the arterial concentration curve of FDG, obtained from Backes et al.^81^, is used for the ${C}_{P}$ concentration profile (Fig. 13). We assumed that ${C}_{P}$ is uniformly distributed throughout the vascular network and will enter into the interstitium^67^. The spatiotemporal distribution of FDG concentrations at seven different post-injection time frames for six considered networks are shown in Fig. 10 and Figs. S6–S8 of Supplementary Material. At the beginning of FDG infusion, ${C}_{i}$ is dominant compared to ${C}_{e}$ and ${C}_{m}$. Over time, the value of ${C}_{i}$ decreases and first ${C}_{e}$ and then ${C}_{m}$ dominate the total concentration (${C}_{tot}={C}_{i}+{C}_{e}+{C}_{m}$). Since the vessels in the capillary network act as source terms for the radiopharmaceutical, the maximum concentration values can be found in the vicinity of the capillary walls. The radiopharmaceutical is then transported and distributed in the other parts of the domain via diffusion and convection mechanisms.

According to the results, the networks induced from the 5 initial sprouts show a more uniform distribution due to the higher MVD. For networks induced from the 3 initial sprouts, due to the heterogeneous structure of the capillary network in tumor region, the radiopharmaceutical delivery to some cells is limited, and as such, uptake is reduced. Furthermore, the maximum total concentration occurs in the tumor region at all time frames due to the high permeability of tumor capillaries compared to normal tissue and also the higher rate of metabolism in cancerous tissue than normal tissue. We also considered the ‘uniform network’ model by assuming uniform solute transport throughout the tumor. In the uniform network model, the vessels do not exist physically. Instead, it is assumed that they are existed in the whole domain as a source term including tumor and normal tissue. The capillaries’ effects have been included in the mathematical modeling by $S/V$ parameter in the equations as a source term in whole the domain (in Eq. S12-1 for concentration transport source term and in Eq. (8) for fluid transport source term); therefore, geometry of parent vessel is not considered in this case. Similarly, ${C}_{P}$ as the injected concentration term, acts as a parameter in the source term equation (in Eq. S12-1 for concentration transport source term)^61,82^. This model can show the effect of the extreme case of MVD on FDG concentration.

**Figure 11:**
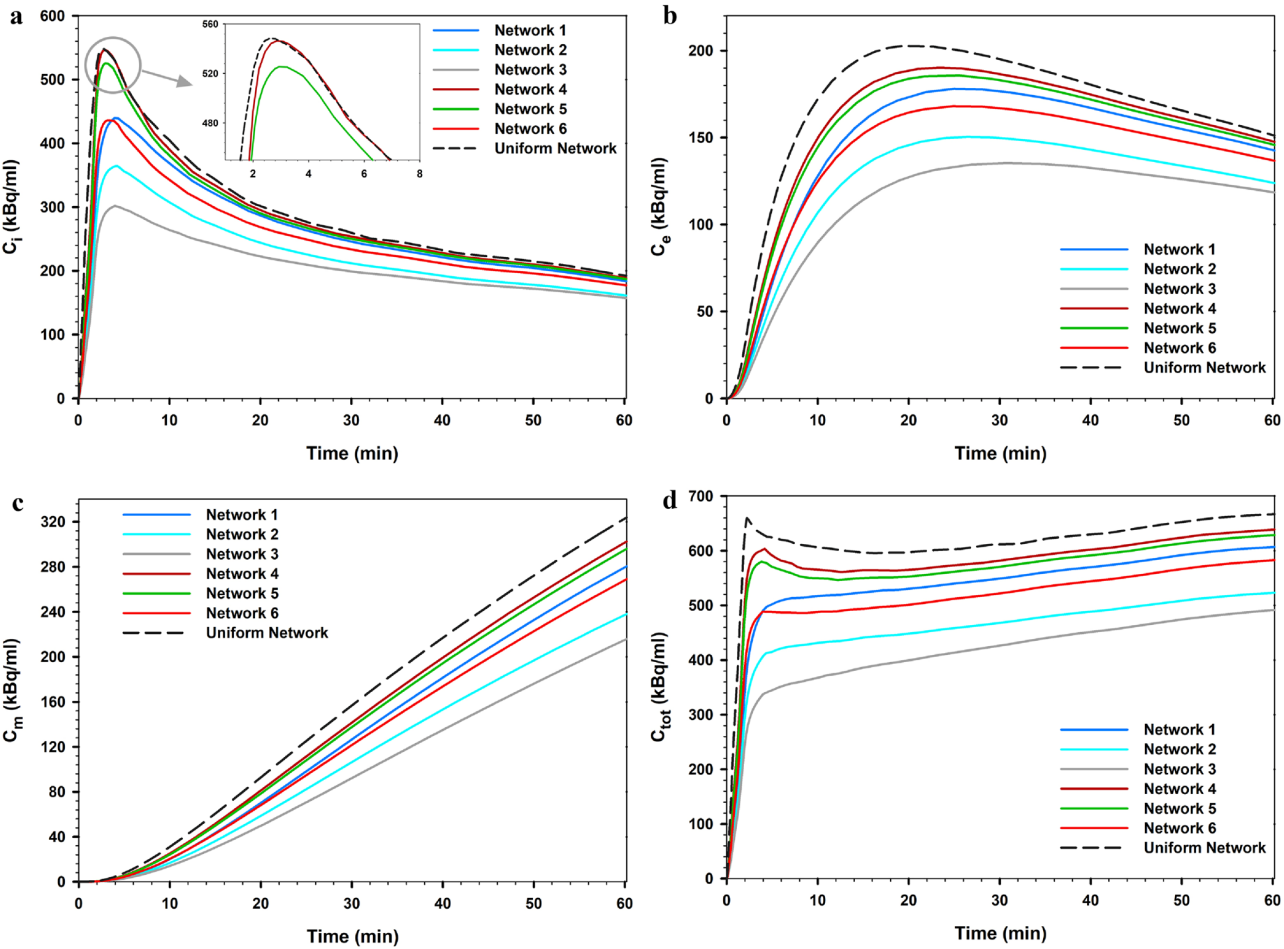
Comparison of the mean FDG concentration in the tumor tissue of all six considered networks calculated via the SDM method and the ‘uniform network’ state. (**a**) ${C}_{i}$, (**b**) ${C}_{e}$, (**c**) ${C}_{m}$, and (**d**) ${C}_{tot}$ (summation of last 3 terms).

Figure 11 shows the mean FDG concentration across the tumor tissue of the considered networks over time. The results include the comparison of outcomes of all six investigated cases with the uniform network case. In addition, the results of FDG distribution were averaged spatially and reported.

**Figure 12:**
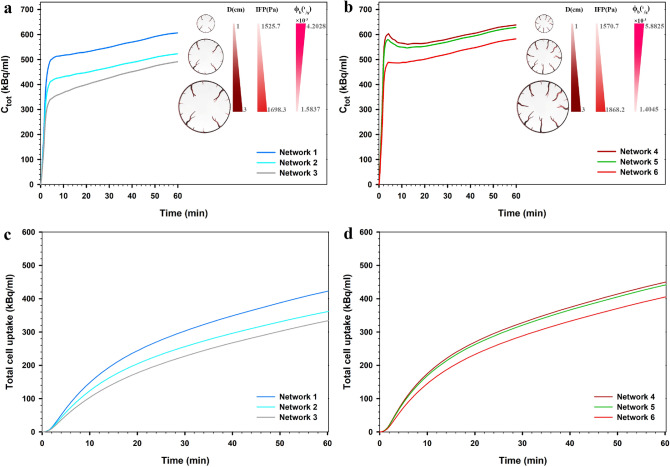
Effect of tumor size on total FDG concentration across the tumor tissue and total cell FDG uptake for the networks of first state considering 3 initial sprouts (**a**,**c**) and the networks of second state considering 5 initial sprouts (**b**,**d**).

Equation (25) in the SDM equations consists of three processes related to diffusion transport ,convection transport, and chemical reactions. The results clearly show that MVD of the capillary network has a significant effect on FDG uptake. The uniform network shows a higher concentration value than the vascularized models. In this case, the solute distribution is only dependent on diffusion from vessels due to the uniform distribution of the capillary network; therefore, the effect of transport in tissue is greatly reduced. At all times, the uptake of the dense network induced from the 5 initial sprouts is greater than that of the 3 initial sprouts, at equal tumor size. By increasing the MVD of networks, the effect of terms related to spatiotemporal transport decreases and the contribution of diffusion terms found a significant effect. When MVD is high, the contribution of diffusion from vessel in the solute transport is greater than diffusion in tissue interstitum; however, with decreasing MVD, the effect of diffusion in tissue interstitum increases. In other words, the diffusion term from vessels depends on the structure of the capillary network. Additionally, as shown in Fig. 12a,b, as the size decreased in tumors by the same MVD, the mean total FDG concentration increased; the contribution of diffusion from vessels increases by decreases in tumor size. Moreover, as shown in the previous section, in the smaller tumor size, the IFP values are lower, leading to an increase in the convection rate of the source term in Eq. (25). Indeed, by reducing the IFP value, the volumetric flow rate from vessels increases, facilitating radiopharmaceutical transport to the tumor tissue. According to Fig. 12c,d, although it appears that the mean intracellular uptake of FDG increased with decreasing tumor size, this is not always fixed for all cases in the clinical conditions and requires consideration of direct effect of cell microscopic variables, such as GLUT expression and HXK enzymes. Consideration of these variables can be used as an advanced model in future efforts to more highlight FDG uptake.

The proposed comprehensive spatiotemporal distribution framework enables the investigation of the relationship between molecular processes and imaging data. Inverse problems can be subsequently developed (future work) to estimate the parameters of interest, including kinetic parameters (K_i_) from dynamic concentration data, diffusion coefficient in the tissue and the input function, towards personalized assessment of disease. Different imaging scenerios (tasks, exam durations, scanners, noise levels, etc.) can be simulated. Subsequently, by constructing appropriate numerical inverse methods (e.g., non-linear regression paradigms), the parameters of interest can be determined. This is hypothesized to result in improved estimates given enhanced SDM modeling over conventional kinetic compartmental methods. Future efforts also include artificial intelligence (AI) towards faster computation of the forward model, and improved inverse models. Such AI-assisted models can be developed via access to large real and synthetic PET datasets.

Overall, combining both the paradigms of therapeutics and diagnostics (i.e., theranostics) into clinical formulations allows for rapid assessment and adjustment of treatment to individual needs for personalized medicine. In theranostics, a diagnostic radiopharmaceutical (e.g. ^68^Ga-PSMA-11 for PET imaging) is used to detect a molecular target with sufficient uptake and abnormal specificity. A therapeutic radiopharmaceutical (e.g., ^177^Lu-PSMA-617) is then administered at a therapeutic dose level to treat the abnormal tissue. Quantitative imaging of the therapeutic radionuclide to image pharmacodynamic response to treatment allows the patient to select personalized and patient-specific dosages to reduce radiation toxicity and optimize the benefits of radiation therapy. Existing radiopharmaceutical therapy practice tends to focus on fixed doses for patients (e.g. 7.4 GBq (200 mCi) for ^177^Lu-DOTATATE). However, to truly personalize therapies it will be necessary to predict the delivered dose. This will require construction of sophisticated digital twins for patients, incorporating more detailed biology and pharmacokinetic modeling. As such, the construction of models such as ours can help enable such a vision, such as our recent efforts^83–85^. While our current model still requires a significant amount of patient-specific data for clinical utility, this study shed lights on the field in what we believe is a needed angle toward a future where deeper understanding on effective factors and parameters translates to more realistic models and decisions. Thus, considering the outcomes of such models, more thorough models incorporating individual patient data will be provided.

### Validation of numerical model

**Figure 13:**
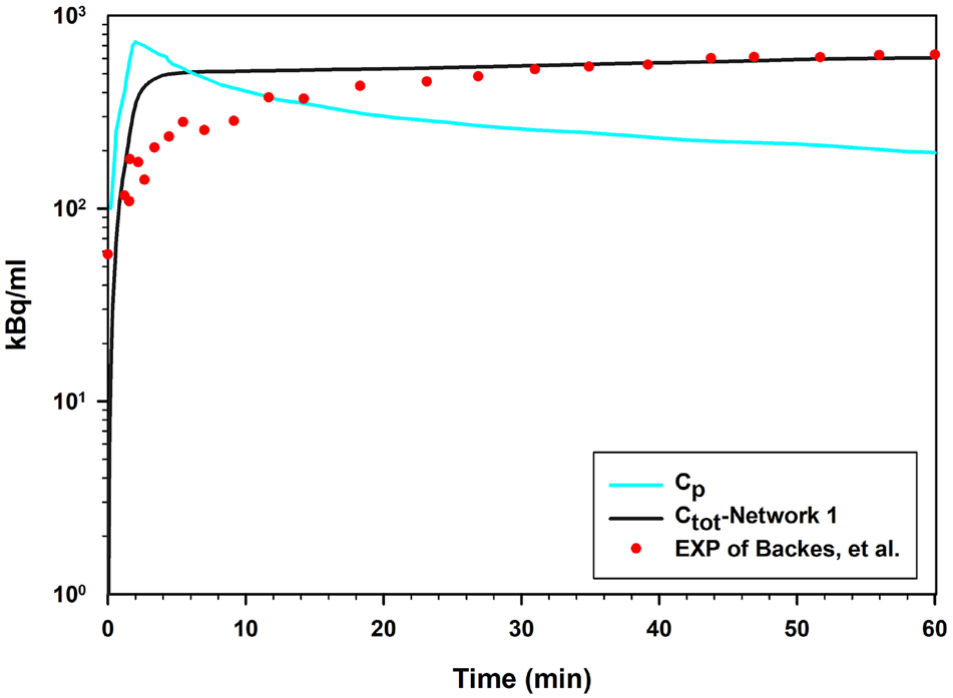
Comparison of results for SDM model with experimental results of Backes et al.^81^ for FDG in tumor region over time.

The distribution trend of FDG uptake in the tissue for ${C}_{e}$ and ${C}_{m}$ agrees well with the experimental results of studies by Carson et al.^86^ and Eastman et al.^87^. Figure 13 shows the comparison of the mean total concentration of FDG in the tumor area with the experimental observation of Backes et al.^81^. Total FDG concentration is calculated in both extracellular and intracellular spaces, as measured in radionuclide imaging. There is a good agreement between the present study and the experimental observations, as the trend of both curves is nearly identical. However, since the domain and conditions of the experimental and modeling are different, the results are not exactly matched. After 15 min, the total concentration of FDG for the current study is very close to the total concentration in the experimental results, and after 25 min, the results exactly matched the experimental results. It should be noted that accurate examination of FDG concentration peaks requires accurate values of the input function at all times. Since the input function of Backes et al. is only available for up to one hour, it is possible to investigate the model's capabilities in distinguishing the kinetic steps of [^18^F]FDG delivery to the extracellular matrix, its transport from the extracellular to the intracellular space, and its intracellular phosphorylation. Additionally, it is possible to evaluate spatiotemporal distribution of radiopharmaceutical by considering different heterogeneity of tumor microenvironment.

## Conclusion

We have presented a comprehensive mathematical model to accurately simulate the spatiotemporal distribution of the [^18^F]FDG radiopharmaceutical uptake within vascularized solid tumors. This model includes the production of tumor-induced microvascular network by a two-dimensional hybrid sprouting angiogenesis method along with the calculation of blood flow in the adaptable new vessels that remodels the network structure in response to metabolic and hemodynamic stimuli. Biological validation of the produced angiogenic networks was performed with in vivo observations. The spatiotemporal distribution of FDG was obtained by coupling intravascular blood flow and interstitial fluid flow equations with the SDM solute transport mathematical model. This model includes convection and diffusion transport mechanisms from the vessels to the tissue and within the tissue, as well as the reaction mechanism.

We demonstrated the capabilities of the model by examining the effects of tumor size and MVD on the radiopharmaceutical distribution over time. Results show that the behavior of FDG radiopharmaceutical distribution is highly dependent on MVD. Due to the high permeability of tumor capillaries, the maximum total concentration occurred in the tumor area at all time frames. Additionally, the maximum concentration value occurs in the vicinity of the vessel walls and decreases with increasing distance from the capillaries due to the low diffusion coefficient. As the MVD increases, the mean FDG uptake into the tumor is also enhanced. In addition, this model correctly predicts the effect of desmoplasia, which involves an increase in IFP in larger tumor sizes. At smaller tumor sizes, an increase in volumetric flow rate, due to lower IFP values, and an increase in the contribution of diffusion mechanism from vessels increased the mean total FDG concentration. The proposed spatiotemporal modeling framework can be used to comprehensively evaluate the effect of various parameters on the spatiotemporal distribution of different radiopharmaceuticals, beyond the usage of conventional methods, including ODE-based kinetic compartment modeling. A goal of the present study is to help bridge the gap among different related fields, including mathematical oncology, radiology, and cancer diagnosis.

## Supplementary Information

Supplementary Information.

## Data Availability

All data used for this study are available from the corresponding author upon request.

## Abbreviations

PET: Positron emission tomography
FDG: Fluorodeoxyglucose
ODE: Ordinary differential equation
PDE: Partial differential equation
SDM: Spatiotemporal distribution model
TME: Tumor microenvironment
mAb: Monoclonal antibodies
ECM: Extracellular matrix
MVD: Microvessel density
TAF: Tumor angiogenesis factor
VEGF: Vascular endothelial growth factor
tEC: Tip endothelial cell
IVP: Intravascular pressure
IFP: Interstitial fluid pressure
IFV: Interstitial fluid velocity
AI: Artificial intelligence
