# Supplementary material for: Spatiotemporal multi-scale modeling of radiopharmaceutical distributions in vascularized solid tumors
Source: Sci Rep. 2022 Aug 26;12:14582. doi: 10.1038/s41598-022-18723-6 (PMC9418261; doi:10.1038/s41598-022-18723-6)
Supplement: Supplementary file 1 — Supplementary Information. [file 41598_2022_18723_MOESM1_ESM.docx]

**Supplementary Material for**

**Spatiotemporal Multi-Scale Modeling of Radiopharmaceutical Distributions in Vascularized Solid Tumors**

Mohammad Kiani Shahvandi^1^, M. Soltani^1,2,3,4,*^, Farshad Moradi Kashkooli^1^, Babak Saboury^5^, Arman Rahmim^6,7^

^1^ Department of Mechanical Engineering, K. N. Toosi University of Technology, Tehran, Iran

^2^ Department of Electrical and Computer Engineering, University of Waterloo, ON, Canada

^3^ Centre for Biotechnology and Bioengineering (CBB), University of Waterloo, Waterloo, ON, Canada

^4^ Advanced Bioengineering Initiative Center, Multidisciplinary International Complex, K. N. Toosi University of Technology, Tehran, Iran

^5^ Department of Radiology, Hospital of the University of Pennsylvania, 3400 Spruce Street, Philadelphia, PA 19104, USA

^6^ Department of Integrative Oncology, BC Cancer Research Institute, Vancouver, BC, Canada

^7^ Departments of Radiology and Physics, University of British Columbia, Vancouver, BC, Canada

^*^ Corresponding author, Email: msoltani@uwaterloo.ca (M. Soltani)

Tel./Fax: +1 (519) 888-4567.

**This file includes:**

Computational approach for angiogenesis model

Fig. S1 and S2

Initial and boundary conditions

Table. S1

Distribution of intravascular pressure

Figs. S3 to S5

PET imaging

Figs. S6 to S8

Table. S2 for Model Parameters

**Computational approach for angiogenesis model**

We selected a $1\times1 {cm}^{2}$ square with $400\times400$ lattice nodes as the computational domain. The set of angiogenesis equations are non-dimensionalized to obtain a suitable form of the governing equations in a $[0, 1]\times[0, 1]$ domain. The obtained equations are discretized by a finite difference scheme to a set of algebraic equations, shown in Eqs. S1-3. The calculations use the probabilistic discretized equations for angiogenesis, which were derived for tip endothelial cells (tEC). The computational lattice of the motion probability distribution around the central cell is shown in Fig. 1. The probabilities of remaining stationary, and right, left, up and down motion for tECs are presented in Eqs. S4-8, respectively. h is the grid size, which is considered equal in both x and y directions, and q specifies the time step. The parameters of the angiogenesis model have been obtained according to the reference values for the concentrations of TAF ($c_{0}$) and fibronectin ($f_{0}$), EC density ($n_{0}$), length ($L$) and time ($\tau$) suggested in reference [1] and are listed in Table S2. For this purpose, non-dimensional parameters are defined as $\tilde{c}=\frac{c}{c_{0}}$, $\tilde{f}=\frac{f}{f_{0}}$, $\tilde{n}=\frac{n}{n_{0}}$, and $\tilde{t}=\frac{t}{\tau}$ where $\tau=\frac{L^{2}}{D_{c}}$ ($D_{c}$ is the TAF diffusion coefficient). For clarity, the tilde ($\sim$) of the parameters is dropped. Solving discretized equations is done in an iterative process. Starting with the initial values for *n*, *f*, and $c$, a new value is obtained at each time step. This value is used as an updated value for the next step and the solution continues until a specified time.

| $n_{i,j}^{q+1}=n_{i,j}^{q}P_{0}+n_{i+1,j}^{q}P_{1}+n_{i-1,j}^{q}P_{2}+n_{i,j+1}^{q}P_{3}+n_{i,j-1}^{q}P_{4}$ | (S1) |
| --- | --- |
| $f_{i,j}^{q+1}=f_{i,j}^{q}[1-\Delta t\gamma n_{i,j}^{q}]+\Delta t\beta n_{i,j}^{q}$ | (S2) |
| $c_{i,j}^{q+1}=c_{i,j}^{q}[1-\Delta t\eta n_{i,j}^{q}]$ | (S3) |

**Migration probabilities for a central tEC:**

| $P_{0}=1-\frac{4\Delta tD_{n}}{h^{2}}+[\frac{\Delta t\chi\left( 1+\alpha c_{i,j}^{q} \right)}{h^{2}}\left( c_{i+1,j}^{q}+c_{i-1,j}^{q}-4c_{i,j}^{q}+c_{i,j+1}^{q}+c_{i,j-1}^{q} \right)-\frac{\Delta t\phi}{h^{2}}\left( f_{i+1,j}^{q}+f_{i-1,j}^{q}-4f_{i,j}^{q}+f_{i,j+1}^{q}+f_{i,j-1}^{q} \right)]$ | (S4) |
| --- | --- |
| $P_{1}=\frac{\Delta tD_{n}}{h^{2}}-\frac{\Delta t}{4h^{2}}\left[ \chi\left( 1+\alpha c_{i+1,j}^{q} \right)\left( c_{i+1,j}^{q}-c_{i-1,j}^{q} \right)+\phi\left( f_{i+1,j}^{q}-f_{i-1,j}^{q} \right) \right]$ | (S5) |
| $P_{2}=\frac{\Delta tD_{n}}{h^{2}}+\frac{\Delta t}{4h^{2}}\left[ \chi\left( 1+\alpha c_{i-1,j}^{q} \right)\left( c_{i+1,j}^{q}-c_{i-1,j}^{q} \right)+\phi\left( f_{i+1,j}^{q}-f_{i-1,j}^{q} \right) \right]$ | (S6) |
| $P_{3}=\frac{\Delta tD_{n}}{h^{2}}-\frac{\Delta t}{4h^{2}}\left[ \chi\left( 1+\alpha c_{i,j+1}^{q} \right)\left( c_{i,j+1}^{q}-c_{i,j-1}^{q} \right)+\phi\left( f_{i,j+1}^{q}-f_{i,j-1}^{q} \right) \right]$ | (S7) |
| $P_{4}=\frac{\Delta tD_{n}}{h^{2}}+\frac{\Delta t}{4h^{2}}\left[ \chi\left( 1+\alpha c_{i,j-1}^{q} \right)\left( c_{i,j+1}^{q}-c_{i,j-1}^{q} \right)+\phi\left( f_{i,j+1}^{q}-f_{i,j-1}^{q} \right) \right]$ | (S8) |

| 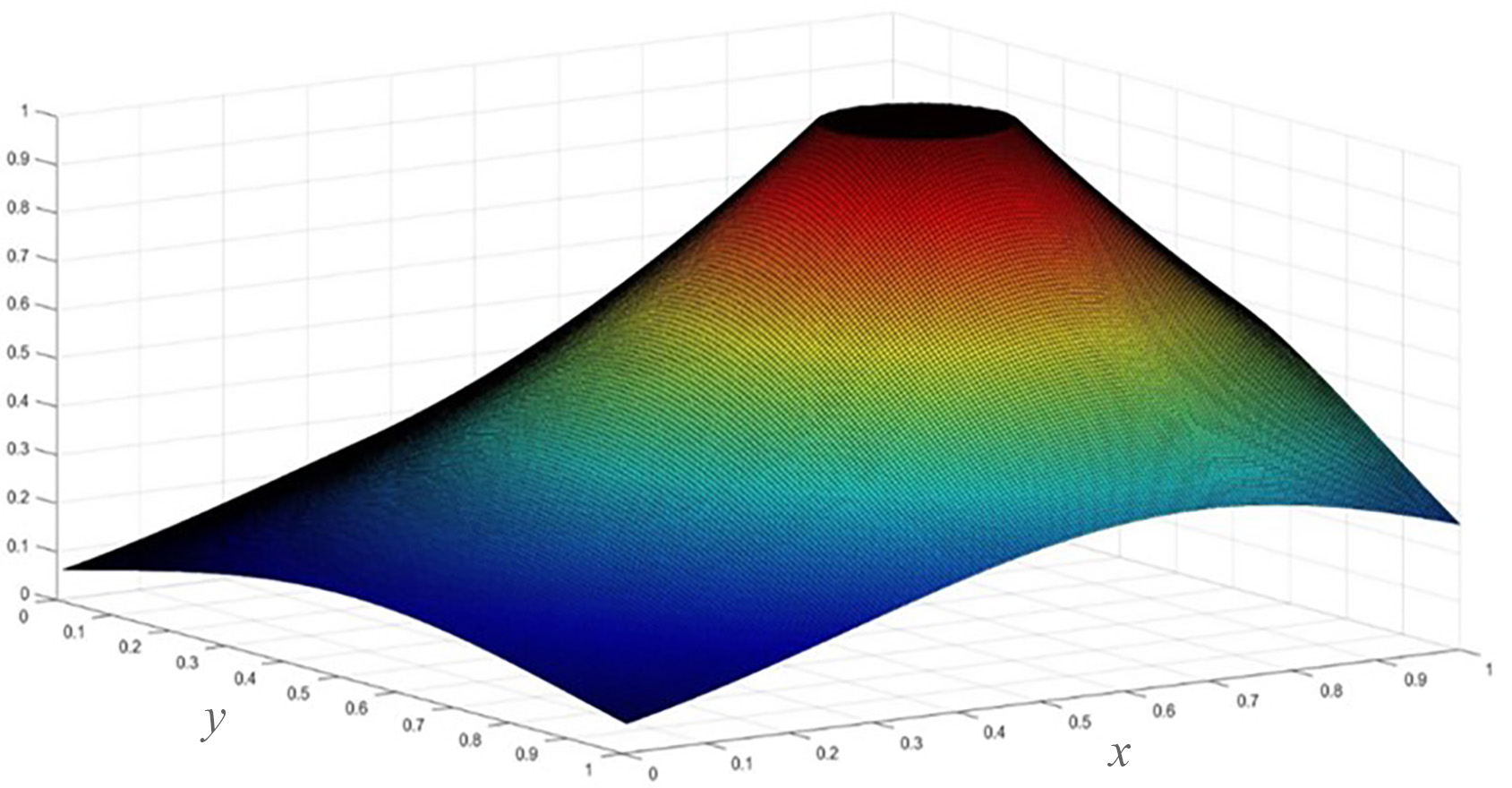 |
| --- |
| **Figure S1.** Initial TAF concentration in the domain. |

| 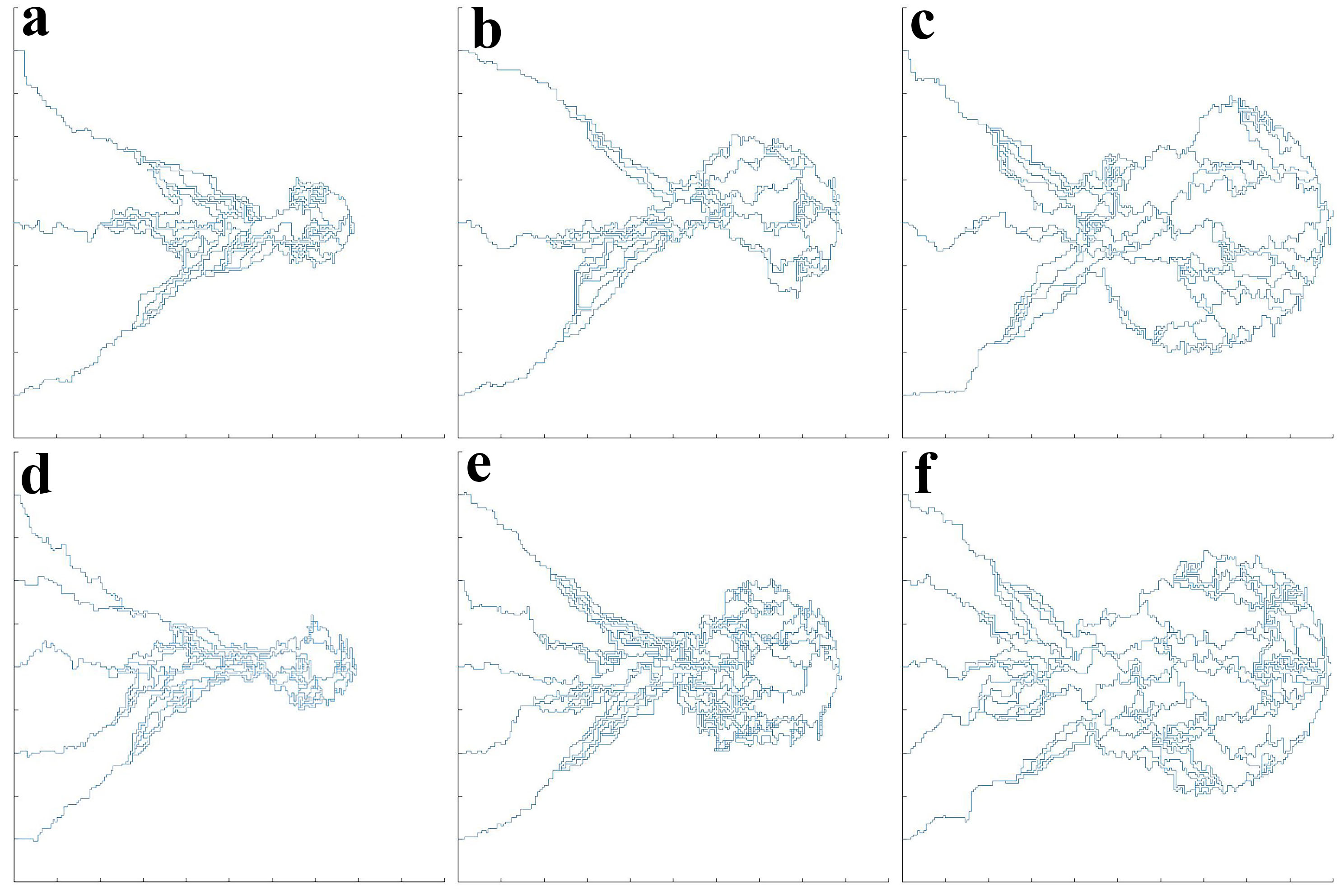 |
| --- |
| **Figure S2.** Different capillary networks generated by sprouting angiogenesis model. The first state considering 3 initial sprouts for (**a**) Network 1 with tumor size $D$, (**b**) Network 2 with tumor size $2\times D$, and (**c**) Network 3 with tumor size $3\times D$; and the second state considering 5 initial sprouts for (**d**) Network 4 with tumor size $D$, (**e**) Network 5 with tumor size $2\times D$, and (**f**) Network 6 with tumor size $3\times D$. |

**Spatiotemporal distribution of FDG:**

| $\frac{\partial C_{i}}{\partial t}=K_{1}C_{P}-\left( K_{2}+K_{3} \right)C_{i}+L_{4}C_{e}$ | (S9) |
| --- | --- |
| $\frac{\partial C_{e}}{\partial t}=K_{3}C_{i}-{(K}_{4}+K_{5})C_{e}$ | (S10) |
| $\frac{\partial C_{m}}{\partial t}=K_{5}C_{e}$ | (S11) |

By combining equations 25, 28 and 29, the following set of equations is obtained. which $Փ_{V}$ and $Փ_{L}$ are the rates of radiopharmaceutical transport per unit volume from blood vessels into the interstitial space, and from the interstitial space into lymph vessels:

| $\frac{\partial C_{i}}{\partial t}=D_{eff}\nabla^{2}C_{i}-v_{i}\cdot\nabla\left( C_{i} \right)+Փ_{V}-Փ_{L}-L_{3}C_{i}+L_{4}C_{e}$ | (S12) |
| --- | --- |
| $Փ_{V}=\frac{S}{V}\left[ L_{P}\left( P_{b}-P_{i}-\left( \pi_{b}-\pi_{i} \right)\sigma\right)\left( 1-\sigma_{f} \right)C_{P}+\frac{Pe}{e^{Pe}-1}P_{m}\left( C_{P}-C_{i} \right) \right]$ | (S12-1) |
| $Փ_{L}=\phi_{L}C$ | (S12-2) |

**Initial and boundary conditions**

To simulate blood flow through the capillary network, $P_{b.inlet}=25$ mmHg (3333 Pa) for inlet pressure and $P_{b.outlet}=10$ mmHg (1333 Pa) for outlet pressure have been selected based on the literature [2-6]. $P_{i}^{0}$ and $C^{0}$ are initialized as zero and the initial blood pressure is considered as $P_{b}^{0}=10$ mmHg (1333 Pa) [7]. The initial diameter of each capillary segment and parent vessel have been considered to be 12 µm and 28 µm, respectively [8, 9]. During the remodeling process, the diameter of parent vessel remains constant and allowable diameter variations for new microvessels is considered in the range of 4µm to 24µm [8, 9]. The boundary conditions of the present study for fluid flow and concentration distribution are reflected in Table S1.

| **Table S1. Boundary conditions used in this study** | | |
| --- | --- | --- |
| Region | Fluid flow | Concentration |
| Boundary between tumor and normal tissues | $\left. -K^{t}\nabla P_{i} \right\vert_{\Omega^{t}}=\left. -K^{n}\nabla P_{i} \right\vert_{\Omega^{n}}$ | $\left. {-D}_{eff}^{t}\nabla C+v_{i}C \right\vert_{\Omega^{t}}=\left. {-D}_{eff}^{n}\nabla C+v_{i}C \right\vert_{\Omega^{n}}$ |
|  | $\left. P_{i} \right\vert_{\Omega^{t}}=\left. P_{i} \right\vert_{\Omega^{n}}$ | $\left. C \right\vert_{\Omega^{t}}=\left. C \right\vert_{\Omega^{n}}$ |
| Outer boundary | $P_{i}$ = 0 | $-n\cdot\nabla C=0$ |
| $\Omega^{t}$ and $\Omega^{n}$ demonstrates the tumor and normal tissue at the boundary, respectively. | | |

**Distribution of intravascular pressure**

| 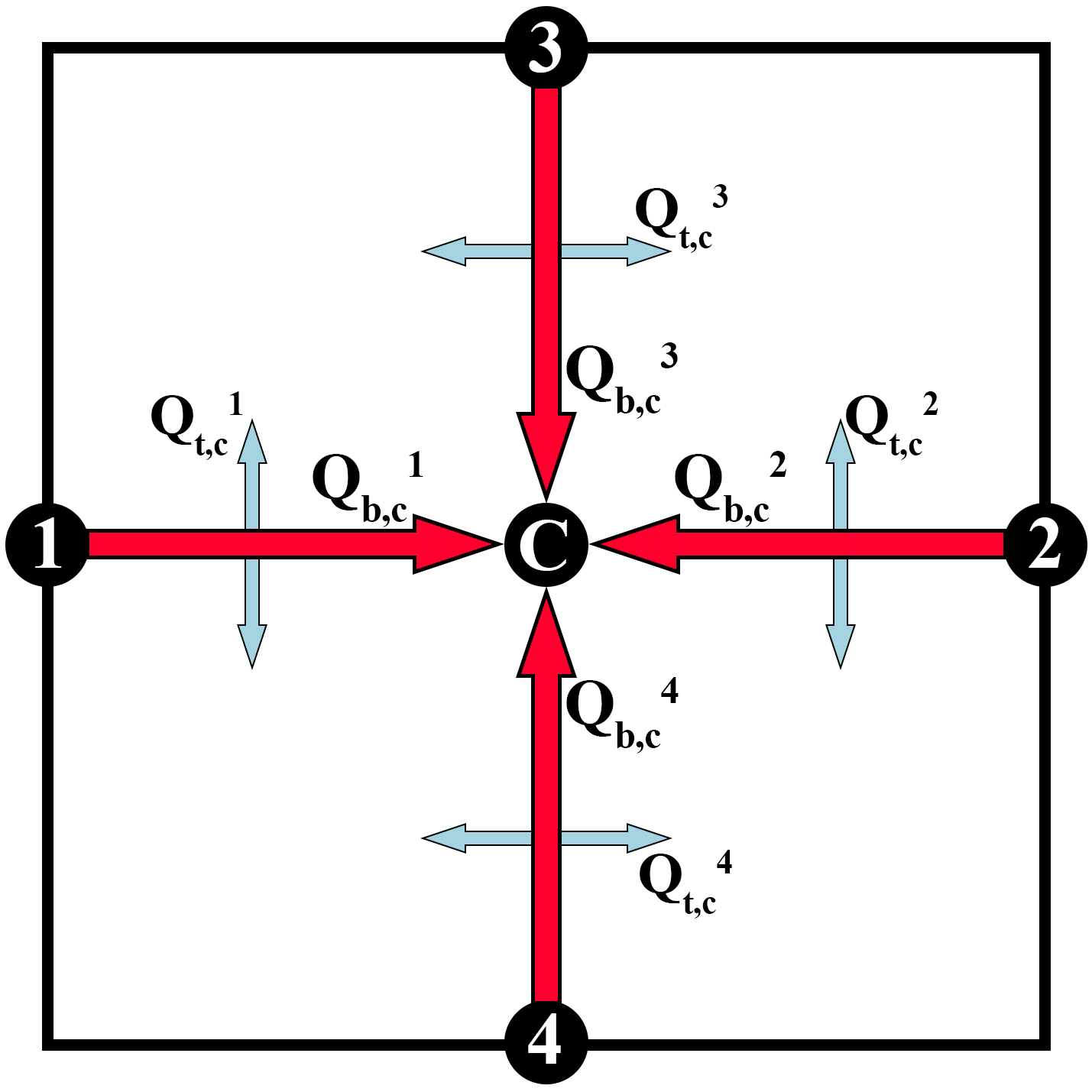 |
| --- |
| **Figure S3.** Schematic of intravascular and transvascular flow in an interconnecting point. It should be mentioned that Microsoft Office PowerPoint 365 was used to create this figure. |

| 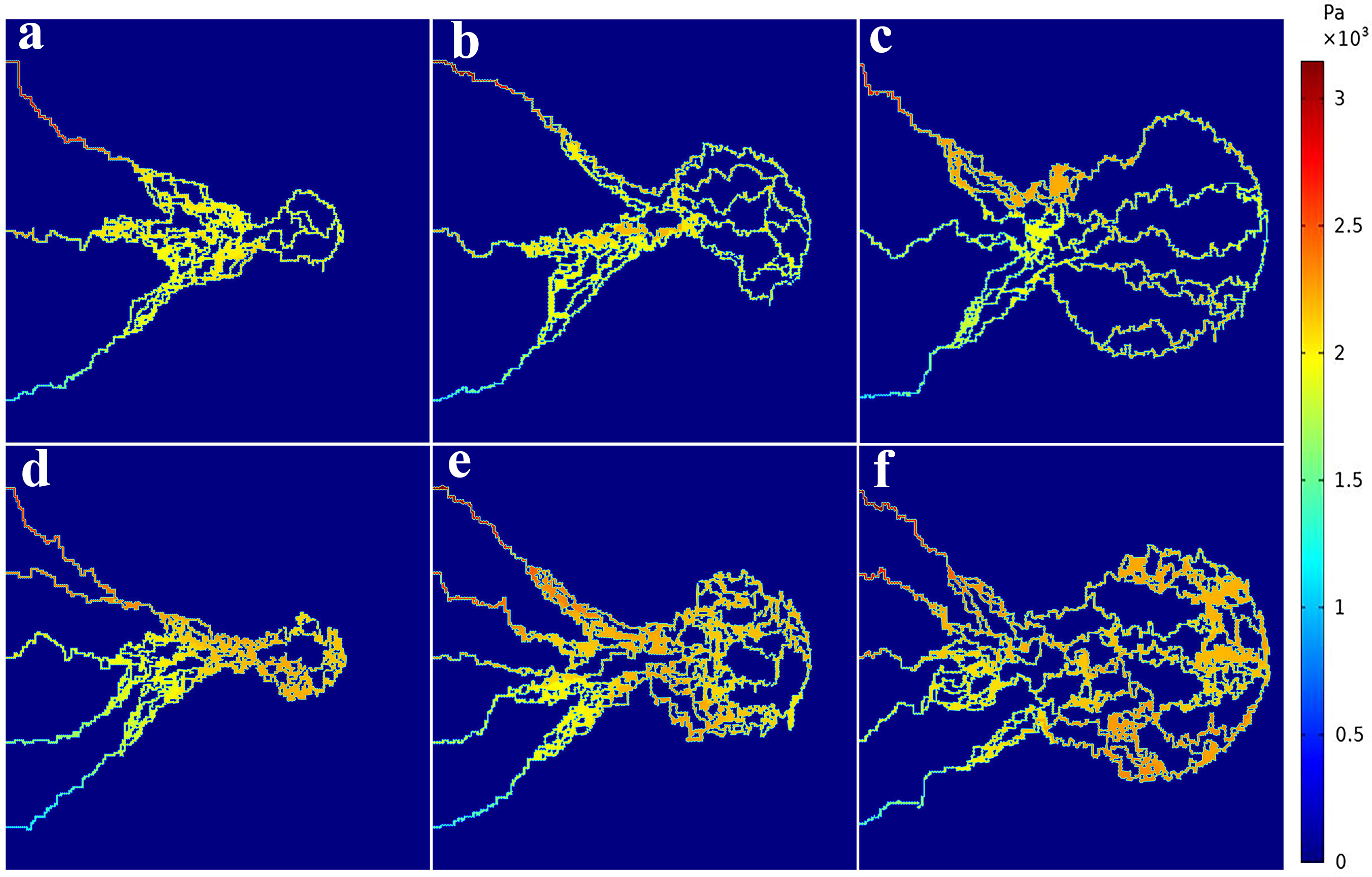 |
| --- |
| **Figure S4.** Intravascular pressure distribution for six considered networks. |

| 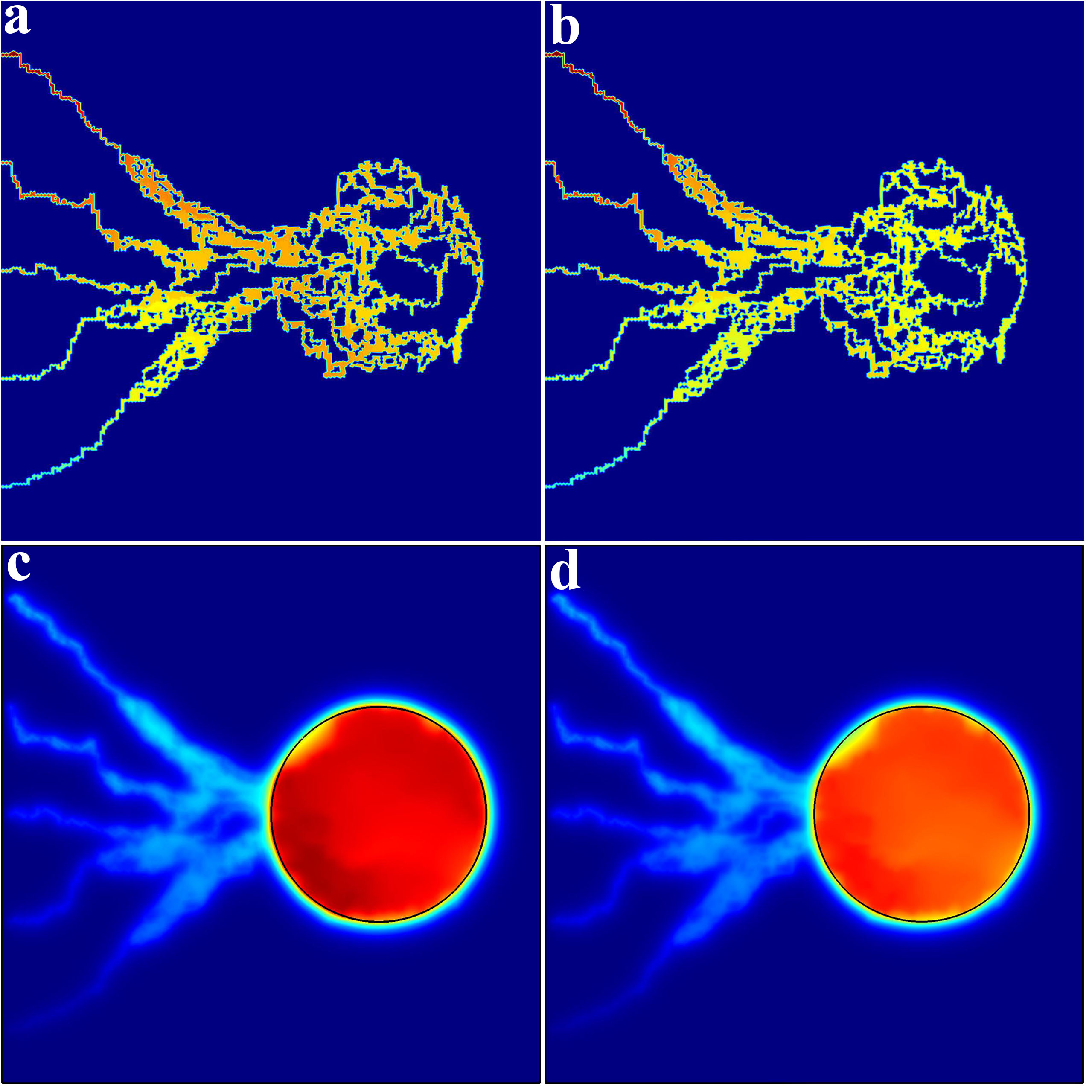 |
| --- |
| **Figure S5.** Comparison of intravascular and interstitial pressures in dynamic (**a, c**) and static (**b, d**) network for network 5. |

**PET imaging**

PET imaging allows the spatial distribution of radiopharmaceuticals to be mapped quantitatively across the body. Hence, quantification of regional uptake of FDG provides complementary information for interpretation of visual image and distinction between benign and malignant lesions. Several techniques with varying complexity for quantitative image analysis include static imaging invoking “standardized uptake values (SUV)” and dynamic imaging referring to simplified radiopharmaceutical kinetic modeling using “Patlak-Gjedde” analysis. By having a time sequence of images, the radioactivity concentration of tissue as a function of time is measured, and the rate of specific biological processes can be determined using appropriate mathematical modeling. Different mathematical models, including conventional compartmental modeling (using ordinary differential equations (ODEs)), and spatiotemporal distribution models (SDMs) (based on partial differential equations (PDEs)), have been developed to simulate solute transport (e.g., drug, nanoparticles, radiopharmaceuticals, etc.) in the tumor microenvironment.

| 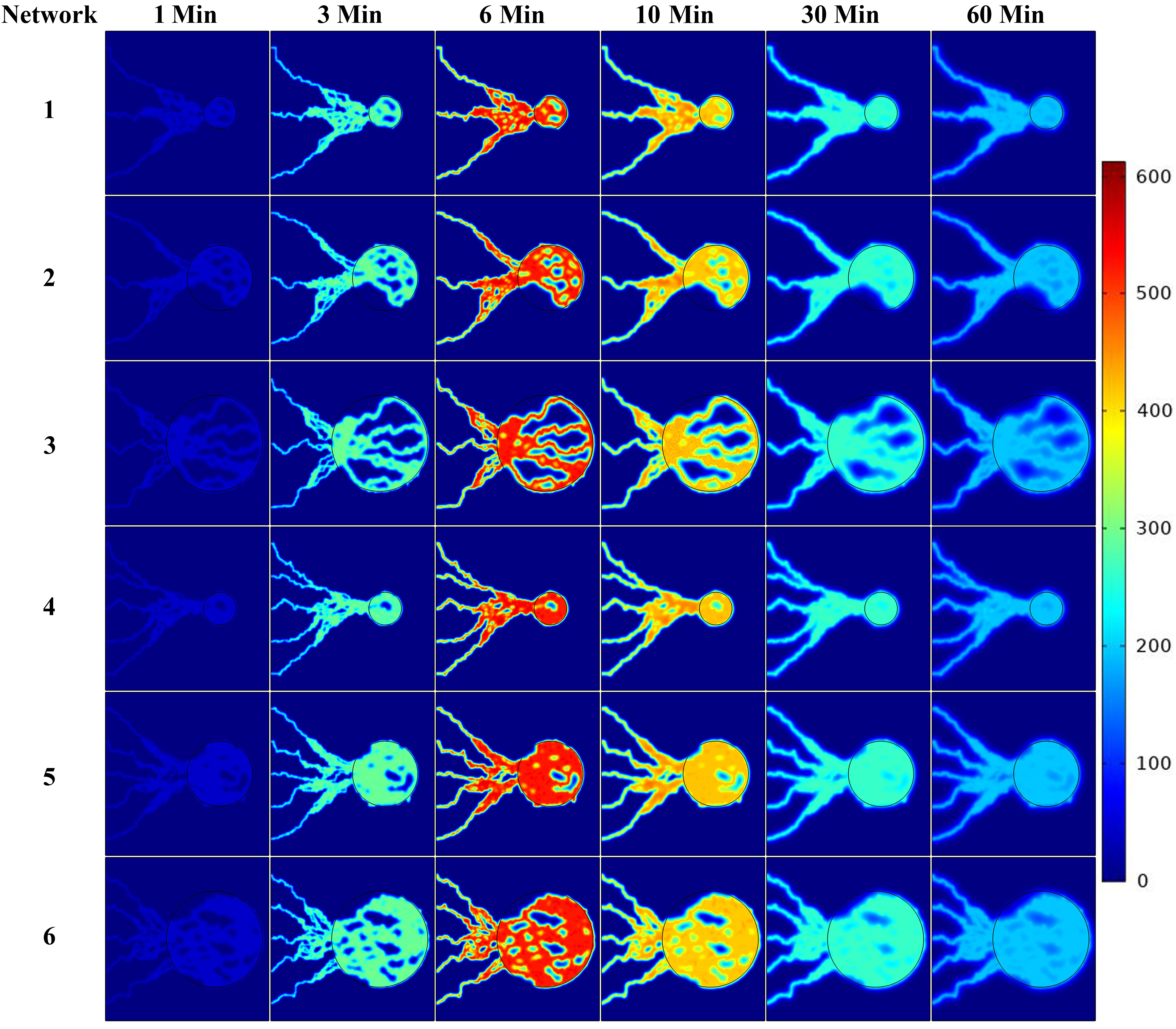 |
| --- |
| **Figure S6.** Spatiotemporal distribution of the extracellular FDG radiotracer concentration ($\frac{kBq}{ml}$) at 1, 3, 6, 10, 30 and, 60 min, in six networks. |

| 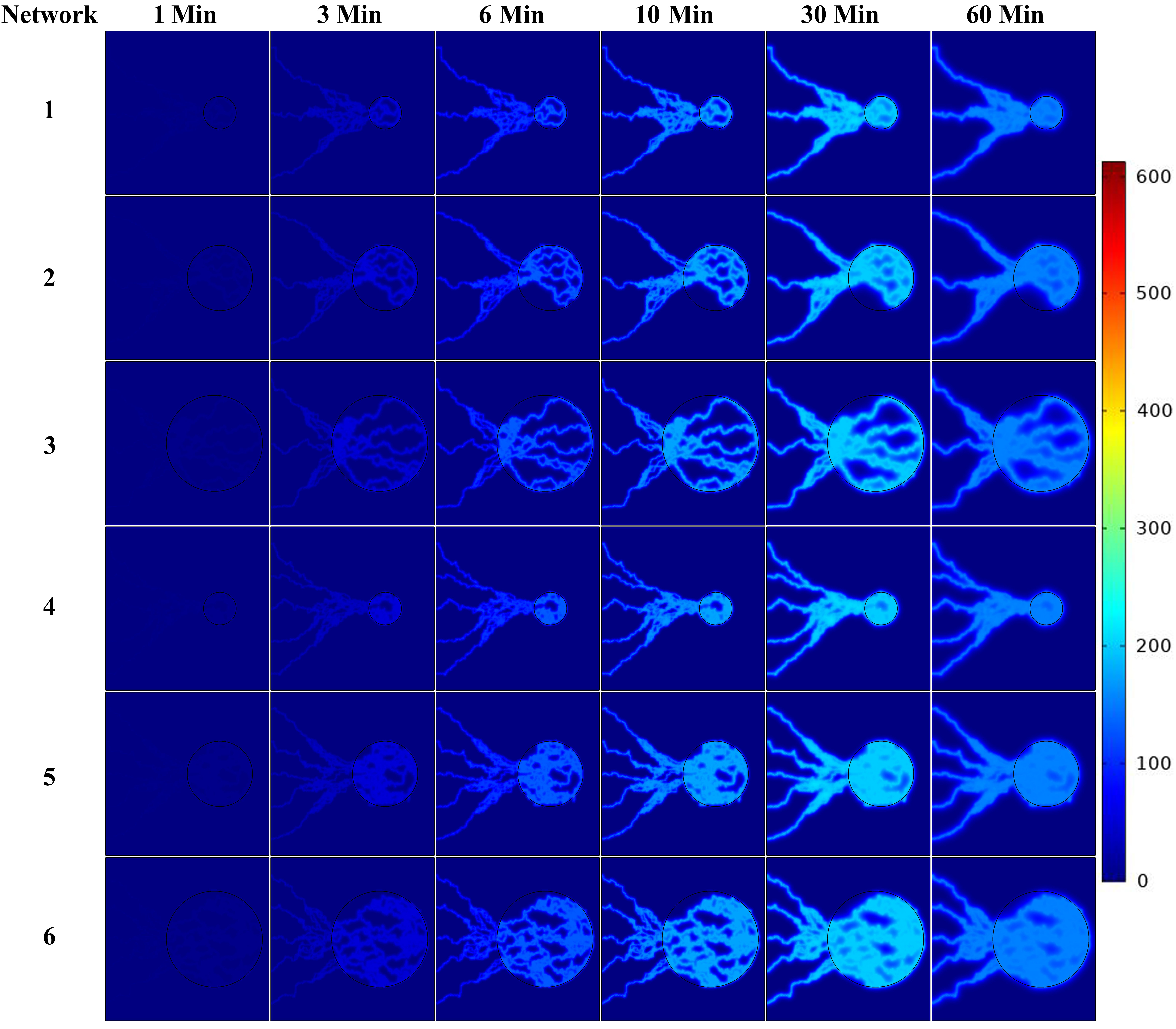 |
| --- |
| **Figure S7.** Spatiotemporal distribution of the intracellular FDG radiotracer concentration ($\frac{kBq}{ml}$) at 1, 3, 6, 10, 30 and, 60 min, in six networks. |

| 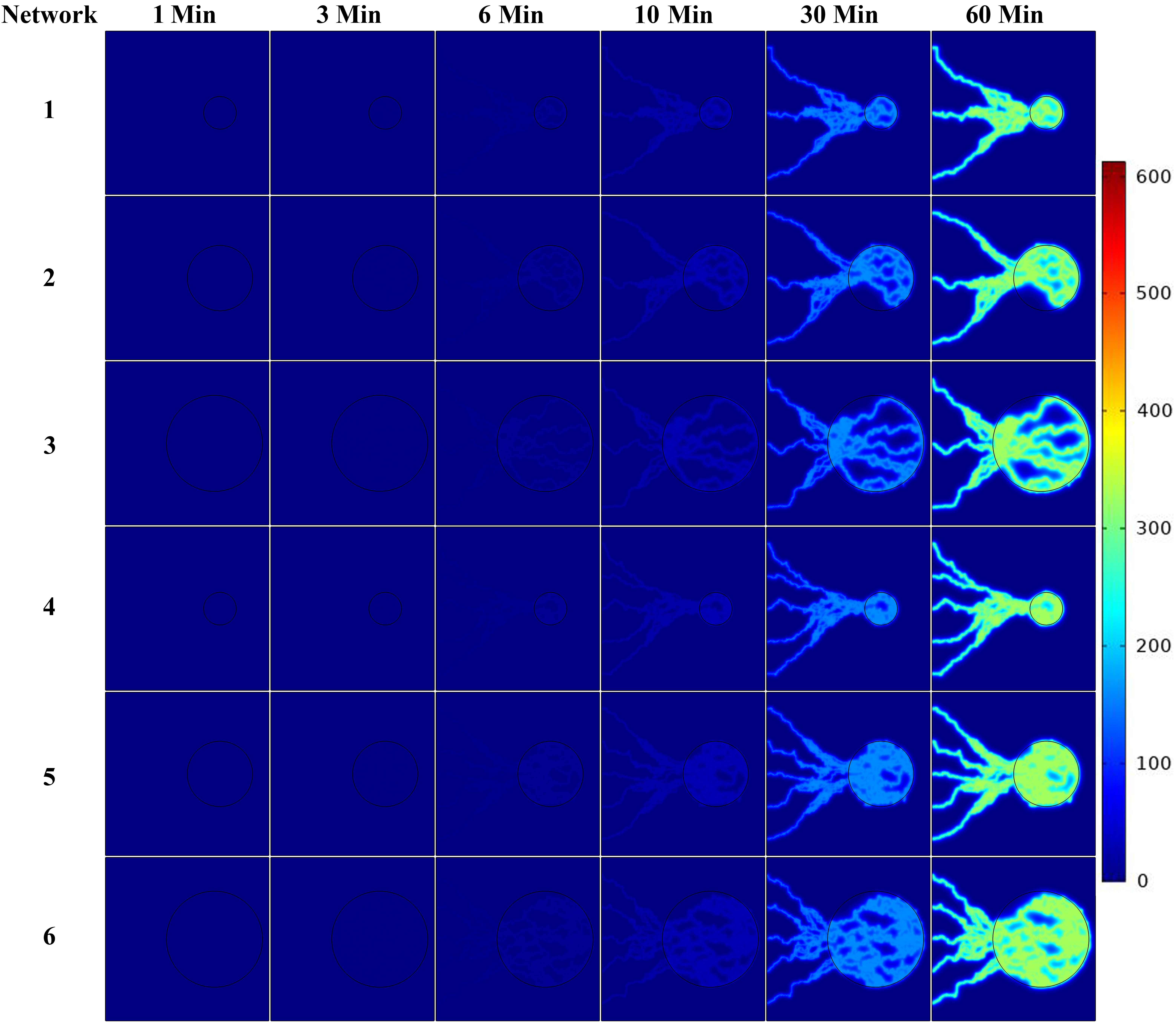 |
| --- |
| **Figure S8.** Spatiotemporal distribution of the 6-phosphate intracellular FDG radiotracer concentration ($\frac{kBq}{ml}$) at 1, 3, 6, 10, 30 and, 60 min, in six network. |

**Model Parameters**

**Table. S2. The parameters used for computational results of the mathematical model.**

| **Parameters** | **Description** | **Value** | **References** |
| --- | --- | --- | --- |
| Angiogenesis | | | |
| $D_{n}$ | Random motility coefficient of EC | 3.5×10^-4^ | [1] |
| $\chi$ | Chemotaxis coefficient | 0.16 | [1] |
| $\alpha$ | Constant of chemotaxis coefficient | 0.6 | [1, 9] |
| $\phi$ | Haptotaxis coefficient | 0.34 | [1] |
| $\beta$ | Production coefficient of fibronectin | 0.01 | [1] |
| $\gamma$ | Uptake coefficient of fibronectin | 0.1 | [1] |
| $\eta$ | Uptake coefficient of TAF | 0.1 | [1] |
| $c_{0}$ | Reference value for concentration of TAF | 1.1×10^-8^ M | [1, 10] |
| $f_{0}$ | Reference value for concentration of fibronectin | 1.36×10^-9^ M | [1] |
| $n_{0}$ | Reference value for concentration of EC density | 10^-10^ M | [1, 11] |
| $D_{c}$ | TAF diffusion coefficient | 2.9×10^-7^ ${cm}^{2}/s$ | [1] |
| Hemodynamics and interstitial fluid flow | | | |
| $K$ | Interstitial hydraulic conductivity | 8.53×10^-9^ ${cm}^{2}/(mmHg\cdot s)$ for healthy tissue | [12, 13] |
|  |  | 4.13×10^-8^ ${cm}^{2}/(mmHg\cdot s)$ for tumor tissue |  |
| $L_{p}$ | Hydraulic conductivity of the microvascular wall | 3.6×10^-8^ $cm/(mmHg\cdot s)$ for healthy tissue | [14, 15] |
|  |  | 2.8×10^-7^ $cm/(mmHg\cdot s)$ for tumor tissue |  |
| $\sigma$ | Average osmotic reflection coefficient | 0.91 for healthy tissue | [8, 16] |
|  |  | 0.82 for tumor tissue |  |
| $\pi_{b}$ | Osmotic pressure of intravascular plasma | 20 $mmHg$ for both healthy and tumor tissues | [8, 16] |
| $\pi_{i}$ | Osmotic pressure of interstitial fluid | 10 $mmHg$ for heathy tissue | [8, 16] |
|  |  | 15 $mmHg$ for tumor tissue |  |
| $S/V$ | Characteristic value of surface area per unit volume of capillaries for mass transport in the interstitium | 7×10^3^ $m^{-1}$ for healthy tissue | [8, 16] |
|  |  | 2×10^4^ $m^{-1}$ for tumor tissue |  |
| $\frac{L_{PL}S_{L}}{V}$ | Lymphatic filtration coefficient | 1.33×10^-5^ $\frac{1}{(pa\cdot s)}$ for healthy tissue | [17] |
| $P_{L}$ | Hydrostatic pressure of lymphatic vessels | 0 $mmHg$ | [17] |
| Hemorheology | | | |
| $U_{cr}$ | Threshold blood velocity ratio of two branches in bifurcations | 2.5 | [18] |
| $\lambda$ | Phenomenological parameter that accounts for the strength of the non-symmetry of the hematocrit distribution at bifurcations | 0.5 | [18] |
| $\mu_{plasma}$ | Dynamic viscosity of plasma | 9×10^-6^ $mmHg\cdot s$ | [19] |
| Remodeling capillary network | | | |
| $\tau_{ref}$ | A positive constant as reference of wall shear stress | 7.73×10^-5^ $mmHg$ | [20] |
| $k_{p}$ | Adaptive response sensitivity of the vessel diameter to changes in intravascular pressure | 0.1 $1/s$ | [9] |
| $k_{m}$ | Proportional coefficient of metabolic stimuli | 0.07 $1/s$ | [9] |
| $k_{s}$ | Shrinking tendency of the vessel in the absence of positive growth stimuli | 0.35 $1/s$ | [9] |
| Spatiotemporal distribution | | | |
| $D_{eff}$ | Effective diffusion coefficient | 0.37×10^-9^ $m^{2}/s$ for healthy tissue | [21, 22] |
|  |  | 1.23×10^-9^ $m^{2}/s$ for tumor tissue |  |
| $\sigma_{f}$ | Filtration reflection coefficient | 0.9 | [23] |
| $P_{m}$ | Microvessel permeability coefficient | 2.26×10^-6^ $m/s$ for healthy tissue | [24] |
|  |  | 7.83×10^-6^ $m/s$ for tumor tissue |  |
| $L_{3}$ | Transport rate parameters into the cell | 8.2×10^-4^ $1/s$ | [25] |
| $L_{4}$ | Transport rate parameters out of the cell | 6.7×10^-4^ $1/s$ | [25] |
| $L_{5}$ | Phosphorylation rate | 5.3×10^-4^ $1/s$ | [25] |

**References**

[1] A.R. Anderson, M.A.J. Chaplain, Continuous and discrete mathematical models of tumor-induced angiogenesis, Bulletin of mathematical biology, 60 (1998) 857-899.

[2] B.W. Wong, E. Marsch, L. Treps, M. Baes, P. Carmeliet, Endothelial cell metabolism in health and disease: impact of hypoxia, The EMBO journal, 36 (2017) 2187-2203.

[3] M. Chaplain, A. Anderson, Mathematical modelling of tumour-induced angiogenesis: network growth and structure, Angiogenesis in Brain Tumors, (2004) 51-75.

[4] Y. Boucher, R.K. Jain, Microvascular pressure is the principal driving force for interstitial hypertension in solid tumors: implications for vascular collapse, Cancer research, 52 (1992) 5110-5114.

[5] C. Voutouri, N.D. Kirkpatrick, E. Chung, F. Mpekris, J.W. Baish, L.L. Munn, D. Fukumura, T. Stylianopoulos, R.K. Jain, Experimental and computational analyses reveal dynamics of tumor vessel cooption and optimal treatment strategies, Proceedings of the National Academy of Sciences, 116 (2019) 2662-2671.

[6] F.M. Kashkooli, M. Soltani, M. Rezaeian, E. Taatizadeh, M.-H. Hamedi, Image-based spatio-temporal model of drug delivery in a heterogeneous vasculature of a solid tumor—Computational approach, Microvascular research, 123 (2019) 111-124.

[7] L.T. Baxter, R.K. Jain, Transport of fluid and macromolecules in tumors. II. Role of heterogeneous perfusion and lymphatics, Microvascular research, 40 (1990) 246-263.

[8] M. Soltani, P. Chen, Numerical modeling of interstitial fluid flow coupled with blood flow through a remodeled solid tumor microvascular network, PloS one, 8 (2013) e67025.

[9] A. Stéphanou, S.R. McDougall, A.R. Anderson, M.A. Chaplain, Mathematical modelling of the influence of blood rheological properties upon adaptative tumour-induced angiogenesis, Mathematical and Computer Modelling, 44 (2006) 96-123.

[10] L. Tang, A.L. Van De Ven, D. Guo, V. Andasari, V. Cristini, K.C. Li, X. Zhou, Computational modeling of 3D tumor growth and angiogenesis for chemotherapy evaluation, PloS one, 9 (2014) e83962.

[11] M. Plank, B. Sleeman, P. Jones, A mathematical model of tumour angiogenesis, regulated by vascular endothelial growth factor and the angiopoietins, Journal of theoretical biology, 229 (2004) 435-454.

[12] F.M. Kashkooli, M. Soltani, M.M. Momeni, A. Rahmim, Enhanced drug delivery to solid tumors via drug-loaded nanocarriers: An image-based computational framework, Frontiers in Oncology, 11 (2021).

[13] F.M. Kashkooli, M. Soltani, M. Rezaeian, C. Meaney, M.-H. Hamedi, M. Kohandel, Effect of vascular normalization on drug delivery to different stages of tumor progression: In-silico analysis, Journal of Drug Delivery Science and Technology, 60 (2020) 101989.

[14] Y. Cai, J. Wu, Z. Li, Q. Long, Mathematical modelling of a brain tumour initiation and early development: a coupled model of glioblastoma growth, pre-existing vessel co-option, angiogenesis and blood perfusion, PloS one, 11 (2016) e0150296.

[15] Y. Cai, J. Zhang, Z. Li, Multi-scale mathematical modelling of tumour growth and microenvironments in anti-angiogenic therapy, Biomedical engineering online, 15 (2016) 685-700.

[16] G. Zhao, J. Wu, S. Xu, M. Collins, Q. Long, C.S. König, Y. Jiang, J. Wang, A. Padhani, Numerical simulation of blood flow and interstitial fluid pressure in solid tumor microcirculation based on tumor-induced angiogenesis, Acta Mechanica Sinica, 23 (2007) 477-483.

[17] G.L. Pishko, G.W. Astary, T.H. Mareci, M. Sarntinoranont, Sensitivity analysis of an image-based solid tumor computational model with heterogeneous vasculature and porosity, Annals of biomedical engineering, 39 (2011) 2360-2373.

[18] T. Alarcón, H.M. Byrne, P.K. Maini, A cellular automaton model for tumour growth in inhomogeneous environment, Journal of theoretical biology, 225 (2003) 257-274.

[19] A.R. Pries, T.W. Secomb, Microvascular blood viscosity in vivo and the endothelial surface layer, American Journal of Physiology-Heart and Circulatory Physiology, 289 (2005) H2657-H2664.

[20] A. Pries, T. Secomb, P. Gaehtgens, Structural adaptation and stability of microvascular networks: theory and simulations, American Journal of Physiology-Heart and Circulatory Physiology, 275 (1998) H349-H360.

[21] H.Ç. Er, A. Erden, N.Ö. Küçük, E. Geçim, Correlation of minimum apparent diffusion coefficient with maximum standardized uptake on fluorodeoxyglucose PET-CT in patients with rectal adenocarcinoma, Diagnostic and Interventional Radiology, 20 (2014) 105.

[22] S.H. Choi, J.C. Paeng, C.-H. Sohn, J.R. Pagsisihan, Y.-J. Kim, K.G. Kim, J.Y. Jang, T.J. Yun, J.-H. Kim, M.H. Han, Correlation of 18F-FDG uptake with apparent diffusion coefficient ratio measured on standard and high b value diffusion MRI in head and neck cancer, Journal of Nuclear Medicine, 52 (2011) 1056-1062.

[23] M. Sefidgar, M. Soltani, K. Raahemifar, H. Bazmara, S.M.M. Nayinian, M. Bazargan, Effect of tumor shape, size, and tissue transport properties on drug delivery to solid tumors, Journal of biological engineering, 8 (2014) 1-13.

[24] C.J. Kelly, M. Brady, A model to simulate tumour oxygenation and dynamic [18F]-Fmiso PET data, Physics in Medicine & Biology, 51 (2006) 5859.

[25] A. Bertoldo, P. Peltoniemi, V. Oikonen, J. Knuuti, P. Nuutila, C. Cobelli, Kinetic modeling of [18F] FDG in skeletal muscle by PET: a four-compartment five-rate-constant model, American Journal of Physiology-Endocrinology and Metabolism, 281 (2001) E524-E536.
